# Ruthenium-Locked Helical Chirality: A Barrier of Inversion and Formation of an Asymmetric Macrocycle

**DOI:** 10.1021/acs.inorgchem.2c02447

**Published:** 2022-09-29

**Authors:** Corjan van de Griend, Johannes J. van de Vijver, Maxime A. Siegler, Remus T. Dame, Sylvestre Bonnet

**Affiliations:** † Leiden Institute of Chemistry, Leiden University, Einsteinweg 55, Leiden 2333CC, The Netherlands; ‡ Small molecule X-ray facility, Department of Chemistry, John Hopkins University, Baltimore, Maryland 21218, United States

## Abstract

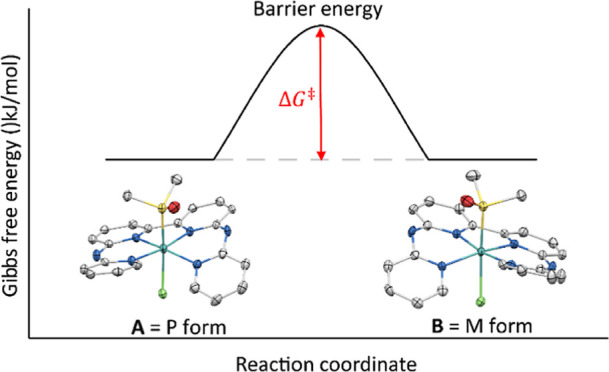

Upon coordination to metal centers, tetradentate ligands based on the 6,6′-bis(2″-aminopyridyl)-2,2′-bipyridine (bapbpy) structure form helical chiral complexes due to the steric clash between the terminal pyridines of the ligand. For octahedral ruthenium(II) complexes, the two additional axial ligands bound to the metal center, when different, generate diastereotopic aromatic protons that can be distinguished by NMR. Based on these geometrical features, the inversion barrier of helical [Ru^II^(L)(RR′SO)Cl]^+^ complexes, where L is a sterically hindered bapbpy derivative and RR′SO is a chiral or achiral sulfoxide ligand, was studied by variable-temperature ^1^H NMR. The coalescence energies for the inversion of the helical chirality of [Ru(bapbpy)(DMSO)(Cl)]Cl and [Ru(bapbpy)(MTSO)(Cl)]Cl (where MTSO is (*R*)-methyl *p*-tolylsulfoxide) were found to be 43 and 44 kJ/mol, respectively. By contrast, in [Ru(biqbpy)(DMSO)(Cl)]Cl (biqbpy = 6,6′-bis(aminoquinolyl)-2,2′-bipyridine), increased strain caused by the larger terminal quinoline groups resulted in a coalescence temperature higher than 376 K, which pointed to an absence of helical chirality inversion at room temperature. Further increasing the steric strain by introducing methoxy groups ortho to the nitrogen atoms of the terminal pyridyl groups in bapbpy resulted in the serendipitous discovery of a ring-closing reaction that took place upon trying to make [Ru(OMe-bapbpy)(DMSO)Cl]^+^ (OMe-bapbpy = 6,6′-bis(6-methoxy-aminopyridyl)-2,2′-bipyridine). This reaction generated, in excellent yields, a chiral complex [Ru(L″)(DMSO)Cl]Cl, where L″ is an asymmetric tetrapyridyl macrocycle. This unexpected transformation appears to be specific to ruthenium(II) as macrocyclization did not occur upon coordination of the same ligand to palladium(II) or rhodium(III).

## Abstract 2

Ruthenium complexes based on the bapbpy ligand form helical chiral complexes due to the steric clash between their terminal pyridyl groups. The coalescence energy for the inversion of this helical chirality was 43 kJ/mol according to variable temperature NMR. Increasing the steric strain by replacing terminal pyridyl groups with quinolyl groups blocked helical interconversion, while introducing *ortho*-methoxy groups resulted in an unexpected ring-closing reaction, forming a dissymmetric macrocycle bound to ruthenium in excellent yields.

## Introduction

The interaction between inorganic compounds and biomolecules such as proteins or nucleic acids has been widely studied^1−7^ since the discovery of the anticancer properties of cisplatin.^8,9^ Especially, the interaction with DNA has gathered wide attention^10,11^ as more and more platinum-based analogues of cisplatin have been reported, with improved properties such as oxaliplatin or satraplatin.^12^ One method to generate specific interaction between inorganic compounds and DNA is to use chiral scaffolds. Octahedral metal complexes have the potential to be chiral, and several synthetic routes have been reported, where one enantiomer of an inorganic compound is enriched or even a single enantiomer is selectively formed.^13,14^ These chiral complexes offer improved characteristics regarding DNA binding, such as higher DNA-binding constants,^15,16^ increased luminescence quantum yields upon binding onto DNA, higher degrees of DNA photocleavage,^17−19^ or improved threading intercalation into DNA.^20^ One notable compound is [Ru(bpy)_2_(dppz)]^2+^, which was originally reported by the group of Barton for its “light switch” properties. This chiral complex is non-emissive in water but becomes luminescent upon intercalation of the dppz moiety into double-stranded DNA.^21^ Later work reported improved luminescence for the delta enantiomer upon binding to mismatch DNA, while the lambda enantiomer showed preference for abasic sites.^22^

**Figure 1:**
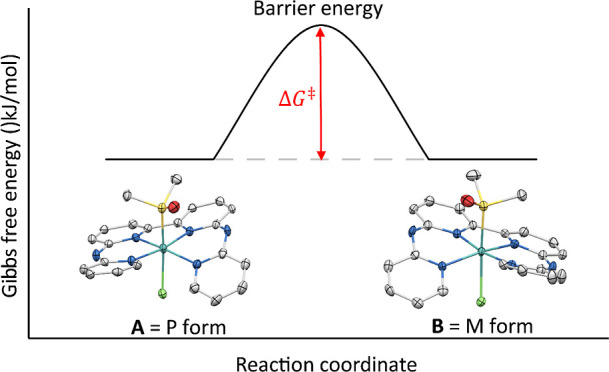
Coalescence energies (Δ*G*^⧧^) for the inversion of the helical chirality of [Ru(bapbpy)(DMSO)(Cl)]^+^.

So far, most chiral complexes discussed in the bioinorganic literature relate to point chirality, where the stereogenic center is either the metal atom itself and/or one of the carbon atoms of a ligand. Other forms of chirality, however, exist. For example, helical chirality offers a fascinating range of compounds known in organic chemistry as helicenes; their properties have been reviewed comprehensively elsewhere.^23,24^ Helicenes consist of ortho-fused aromatic rings that cannot adopt a flat planar conformation due to the steric hindrance of the terminal rings; these molecules therefore adopt a helical structure, which is inherently chiral and can exist as two enantiomers noted P and M.^25^ The inversion barrier between these two forms rapidly increases upon extension of the aromatic system with activation energies of 96.3 kJ/mol for [5]helicine^26^ and 151.5 kJ/mol for [6]helicine.^27^ A recent trend in this field is the coordination of helicene-containing ligands to metal centers, which has been shown to alter the properties of the helicenes.^28−30^ For example, the coordination of helical ligands to iridium compounds resulted in a light-green phosphorescence with unusually long lifetimes. Recently, the group of Crassous reported the synthesis and structural characterization of a range of helicene-like ligands coordinated to ruthenium, forming metal complexes based on the [Ru(bpy)_3_]^2+^ scaffold but with an extended π conjugation system.^31^ The same group even reported the synthesis and crystal structure of an enantio-enriched binuclear ruthenium complex linked by a helical ligand containing two bpy-like moieties. Another type of helical chiral metal complexes are those based on the non-chiral 6,6′-bis(2″-aminopyridyl)-2,2′-bipyridine ligand (bapbpy).^32^ Upon metal coordination, this type of ligands can no longer adopt a flat conformation due to the steric clash between its terminal pyridines, which imposes a helical conformation to the tetrapyridyl structure (Figure 1). Typically, single-crystal X-ray structures of these helicenoid complexes show both helical enantiomers present in the crystal lattice.^31^ As we recently found that bapbpy-based ruthenium^33,34^ or platinum^35^ complexes can interact with DNA and considering that the ease at which chiral inversion of the helix occurs had remained unknown up to now, we engaged in this work into determining the barrier of inversion of the helical chirality of such molecules.

**Figure 2:**
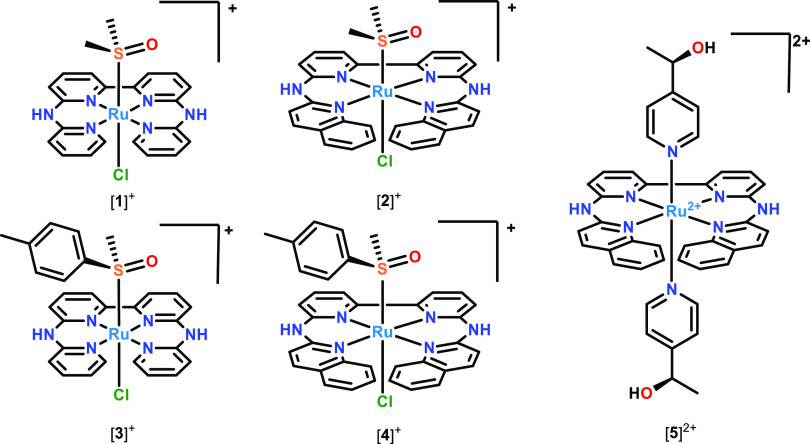
Chemical structure of the compounds used in the helical inversion ^1^H NMR study.

Considering that the helicity of these complexes is a direct consequence of the steric strain between the terminal pyridyl groups of the bapbpy ligand, we synthesized ruthenium(II) bapbpy-based derivatives with various levels of steric strain on these terminal pyridines (Figure 2) and studied the rate of interconversion between the two helical enantiomers using variable-temperature ^1^H NMR. We explored this helical inversion both on chiral complexes of the type [Ru(L)(DMSO)Cl]Cl ([**1**]Cl and [**2**]Cl, with L = bapbpy or L = 6,6′-bis(aminoquinoline)-2,2′-bipyridine = biqbpy, respectively), where DMSO is the non-chiral ligand dimethylsulfoxide, and on their analogues [Ru(L)(MTSO)Cl]Cl ([**3**]Cl-[**4**]Cl with L = bapbpy and biqbpy, respectively) and [Ru(biqbpy)(EtOHPy)_2_](PF_6_)_2_ ([**5**](PF_6_)_2_), where MTSO and EtOHPy are the enantiomerically pure chiral ligands (*R*)-methyl *p*-tolylsulfoxide and (*R*)-(+)-α-methyl-4-pyridinemethanol, respectively. In the latter cases, the enantiopure nature of the axial ligand(s) generated diastereomers upon coordination to the helical chiral complexes that could be distinguished by NMR. Finally, we report the serendipitous discovery of a macrocyclization reaction taking place when the most sterically hindered ligand of the series, L = 6,6′-bis(6-methoxy-aminopyridyl)-2,2′-bipyridine (OMe-bapbpy), was coordinated to ruthenium(II).

## Results

### Synthesis

The known achiral tetradentate ligands bapbpy and biqbpy were reacted with the precursor [Ru(DMSO)_4_Cl_2_] to form racemic mixtures of the chiral complexes [**1**]Cl and [**2**]Cl, respectively. This reaction was achieved by a straightforward overnight reflux at 80 °C in ethanol to afford the products in 68 and 96% yield, respectively. The DMSO axial ligand was further substituted with the enantiomeric pure sulfoxide ligand MTSO to afford mixtures of diastereoisomer complexes of compounds [**3**]Cl and [**4**]Cl, respectively. To complete our investigations on the chirality of these structures, we finally substituted both axial ligands with chiral pyridine EtOHPy to afford compound [**5**](PF_6_)_2_ as a mixture of epimers (*P*,*R*,*R* and *M*,*R*,*R*) that differ in only one stereo-center.

**Figure 3:**
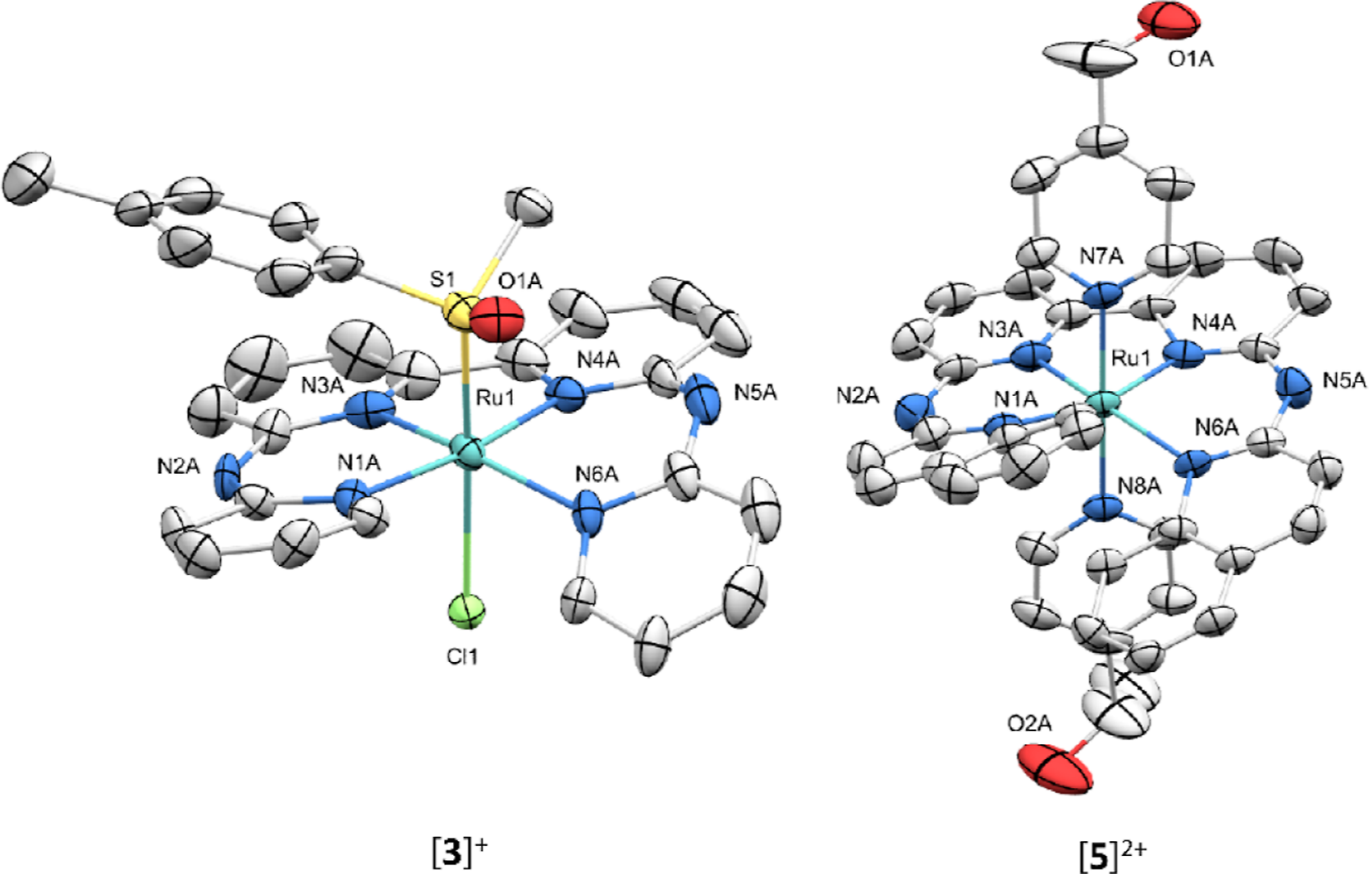
Displacement ellipsoid plots (50% probability level) for [**3**]PF_6_ and [**5**](PF_6_)_2_ at 110(2) and 173(2) K, respectively. Counterions and hydrogens have been omitted for clarity.

Single crystals suitable for X-ray structure determination for [**3**]PF_6_ and [**5**](PF_6_)_2_ were obtained by vapor diffusion of diethylether into a methanol solution containing the metal compound (0.2 mg/mL), in the presence or in the absence, respectively, of a drop of 55% HPF_6_ in water. The crystal structures are shown in Figure 3 and a selection of bond lengths and angles is reported in Table 1. Both structures show the chiral helically distorted conformation of the tetradentate ligand. The crystal lattice of [**3**]PF_6_ contained both the (*P*,*R*) and (*M*,*R*) diastereomers and that of [**5**](PF_6_)_2_ contained both the (*P*,*R*,*R*) and (*M*,*R*,*R*) epimers (Figure S1). The N1N3N4N6 dihedral angle, which is one measure of the helical distortion, was 9.9(6)° for [**3**]^+^ and 16.6(4)° for [**5**]^2+^_._ Clearly, the extended aromatic system of the biqbpy ligand, compared to bapbpy, resulted in an increase of in helical distortion of the ligand upon coordination to ruthenium(II).

**Table 1:** Selected Bond Distances (Å) and Angles (°) Found in the Crystal Structures of [**3**]PF_6_, [**5**](PF_6_)_2_, [**6**]OTf, [**7**](OTf)·MeOH, and [**8**](OTf)_2_

compound	[**3**]^+^^b^	[**5**]^2+^^c^	[**6**]^+^^b^	[**7**]^+^	[**8**]^2+^^b^
M–N1	2.098(9)	2.146(8)	2.089(7)	2.123(9)	2.078(2)
M–N3	2.031(9)	2.023(8)	2.033(6)	2.027(10)	2.065(2)
M–N4	2.022(8)	2.020(8)	2.031(7)	2.012(9)	1.991(2)
M–N6	2.102(9)	2.180(8)	2.051(6)	2.089(11)	1.977(2)
M–S1	2.207(2)		2.214(2)	2.192(3)	
M–Cl1	2.430(2)		2.421(2)	2.456(3)	
C1–O2–C20			124.9(7)	127.1(10)	
C5–N2–C6	135.5(9)	132.0(5)	134.9(7)	136.6(10)	130.3(3)
C15–N5–C16	130.3(3)	132.5(9)	127.8(7)	131.6(11)	132.5(3)
N1–M–N4	172.5(3)	165.3(3)	171.2(3)	168.6(4)	162.15(10)
N3–M–N6	165.1(3)	167.7(3)	169.8(3)	173.4(8)	165.34(10)
τ_4_^a^	0.1(6)	0.1(9)	0.1(3)	0.1(3)	0.2(3)
distortion	10.8(4)	16.6(4)	1.2(2)	1.4(0)	20.8(9)

a The coordination angles N1–M1–N4 and N3–M1–N6 were used to calculate τ_4_.^39^
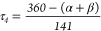

b Consists of two crystallographically independent formular units for the structure. The bond distance and angles are given for molecule A.

c Consists of four crystallographically independent formular units for the structure. The bond distance and angles are given for molecule A.

**Figure 4:**
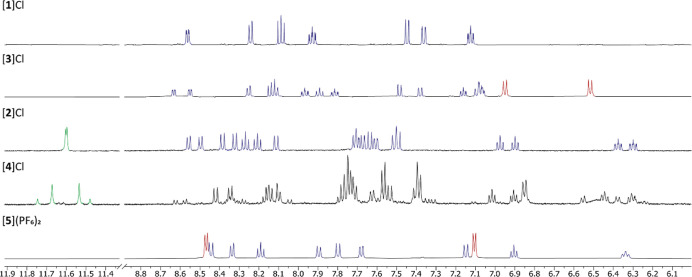
^1^H NMR spectra of [**1**]^+^ and [**3**]^+^ in methanol-*d*_4_, [**2**]^+^ and [**4**]^+^ in DMSO-*d*_6_ and [**5**]^2+^ in acetone-*d*_6_. The blue-colored peaks are assigned to the tetradentate ligands, the red peaks are assigned to the chiral ligand, the green peaks are assigned to the bridging amine, and the black peaks are unassigned.

The room-temperature ^1^H NMR spectra of the ruthenium complexes already offer an intriguing insight into their chiral structure and dynamics (Figure 4). The helical chiral, racemic complex [**1**]Cl showed only seven aromatic signals, indicating that on the NMR time scale, this complex has an average plane of symmetry perpendicular to the average plane of the babpbpy ligand. By contrast, upon substitution of the non-chiral axial DMSO ligand with the chiral, enantiomerically pure MTSO ligand, all aromatic signals assigned to the bapbpy ligand were doubled in [**3**]Cl, which was consistent with the formation of diastereotopic protons, while the two aromatic MTSO signals indicate the presence of only one species. Overall, the doubling of the babpy-based proton peaks in [**3**]^+^ can be attributed to the loss of the plane of symmetry in the complex concomitant with the substitution of the achiral sulfoxide DMSO with the chiral MTSO. The fact that one single species is observed in the room-temperature ^1^H NMR spectrum of [**3**]Cl, while its crystal structure contains both diastereoisomers, leads to the conclusion that the helicity inversion due to the switching of the position of the terminal pyridines is rapid on NMR time scales at room temperature, rendering the separation of these diastereomers impossible.

Interestingly, the biqbpy analogue [**2**]Cl showed 18 aromatic signals at room temperature in solution, indicating that, unlike [**1**]Cl, this complex has no average plane of symmetry: the chemical environment of the terminal quinolines on the side of the DMSO versus chloride axial ligands (i) is different enough to be distinguished and (ii) cannot exchange on the NMR time scale at room temperature. In other words, each pair of protons of the biqbpy ligand that are equivalent by symmetry in the free ligand, and would remain equivalent in a hypothetical planar conformation tetracoordinated to a metal center, becomes diastereotopic in the real, helical complex. In the absence of rapid exchange of the helicity of the complex, they can be distinguished by NMR. Biqbpy has hence increased strain, compared to bapbpy, with regard to helix inversion, which can be interpreted as a cause of the larger size of the quinoline groups, compared to pyridine groups in [**1**]^+^. The substitution of the achiral DMSO ligand in [**2**]^+^ with the chiral sulfoxide MTSO, to give [**4**]^+^, led according to ^1^H NMR to the formation of one major and one minor diastereoisomer, with a diastereoisomeric excess of ∼50. The symmetry here was very low as well, which pointed to the absence of inversion of the helix at room temperature. For example, four amine peaks can be clearly distinguished near 11.6 ppm. As a note, diastereomers are distinct species with a priori different physical properties that one expects to be separable on achiral high-performance liquid chromatography (HPLC) columns. Disappointingly, we were unable to separate these two diastereoisomers, neither on an achiral preparative nor on a chiral semi-prep Astec CYCLOBOND I 2000 DMP column. The labile chloride ligand proved problematic as it was rapidly hydrolyzed with the aqueous component of the HPLC eluents and further substituted with MeCN, effectively rendering any eluents containing H_2_O or MeCN dysfunctional. Other eluent systems were tried but did not yield the desired separation. Still, we were able to identify two species when an analytical sample was measured on the chiral semi-prep DMP column, with 0.1 M NH_4_Cl in MeOH as an eluent, as seen in Figure S2. Finally, in the NMR spectrum of [**5**](PF_6_)_2_ (Figure 4), only nine peaks were assigned to the biqbpy ligand and two peaks for the axial pyridines (EtOHPy) as the complex has a C2 rotational symmetry axis. Each molecule has three chiral centers, and the crystal structure shows the presence of both the P and M epimers. Although at a first glance the ^1^H NMR seemed to show only one species in the aromatic region, in the aliphatic region, it showed two doublets overlapping at 1.09 ppm, which are assigned to diastereomeric methyl groups of the coordinated EtOHPy. The ^13^C NMR spectrum also showed two peaks almost overlapping at 65.7 ppm for the CH chiral carbon atom of the axial ligands. These results confirm the formation of two closely related epimers, even though the chiral centers are too far apart to directly influence each other, and most aromatic signals for the epimers seem to overlap. Also, in this case, the separation of these two epimers could not be achieved.

**Figure 5:**
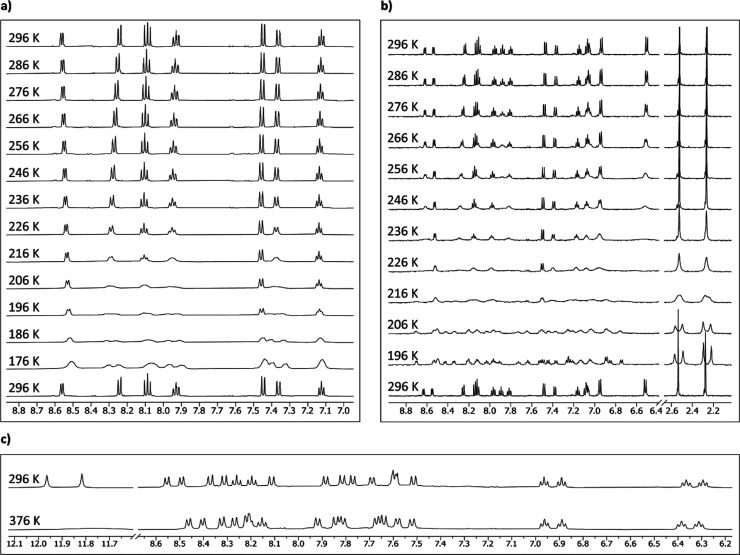
(a) Variable-temperature ^1^H NMR of [**1**]^+^ in methanol-*d*_4_ from 296 to 176 K. (b) [**3**]^+^ in methanol-*d*_4_ from 296 to 196 K. (c) [**2**]^+^ in DMSO-*d*_6_ from 296 to 376 K.

Clearly, upon coordination to the metal, the helicity of the bapbpy ligand switches back and forth quickly at room temperature, while that of biqbpy is blocked. To measure the inversion barriers for both types of structures, variable-temperature ^1^H NMR spectra were recorded for compounds [**1**]^+^, [**2**]^+^, and [**3**]^+^ (Figure 5). The spectra of [**1**]^+^ and [**3**]^+^ showed a doubling of the number of peaks as the temperature decreased. Thus, as the temperature was lowered, the interconversion of both terminal pyridines in the helical complexes became slow, compared to NMR time scales, leading to vanishing of the average plane of symmetry of the bapbpy ligand in both enantiomers and to the observation of 1:1 pairs of diastereoisomeric bapbpy protons for compound [**1**]^+^. For compound [**3**]^+^, the same phenomenon resulted in the blocking of the interconversion of the two diastereomers, below which their protons became distinguishable as well. The integrals of both diastereoisomers were roughly 1:1, showing that these diastereomers had similar Gibbs free energies. The coalescence energy of [**1**]^+^ was found to be 43 kJ/mol, determined from the doublet at 8.25 ppm and at a corresponding coalescence temperature of 206 K.^36^ For compound [**3**]^+^, 44 kJ/mol was found from the singlet at 2.5 ppm and at a coalescence temperature of 216 K. Both values are significantly lower than the 96.3 kJ/mol reported for [5]helicine, demonstrating that comparatively very rapid interconversion occurs at room temperature. The contribution of the different sulfoxide was minimal, which indicated that the coalescence energy was mainly determined from the size of the pyridyl group and not from the size of the sulfoxide substituents.

For compound [**2**]^+^, the coalescence temperature was dramatically increased, compared to that of [**1**]^+^ and [**3**]^+^. When the temperature was increased up to 376 K, which was the limit of our spectrometer, 18 diastereotopic protons could still be seen, pointing to an absence of exchange of the terminal quinolines even at such high temperatures. Overall, the increased size of the quinolyl groups in [**2**]^+^ resulted in a significant increase in the coalescence energy of this complex, compared to that of [**1**]^+^. This coalescence energy must be higher than 79 kJ/mol, which is the value calculated based on the chemical shift of the two triplets at 6.37 and 6.29 ppm and at a coalescence temperature of 376 K.

**Figure 6:**
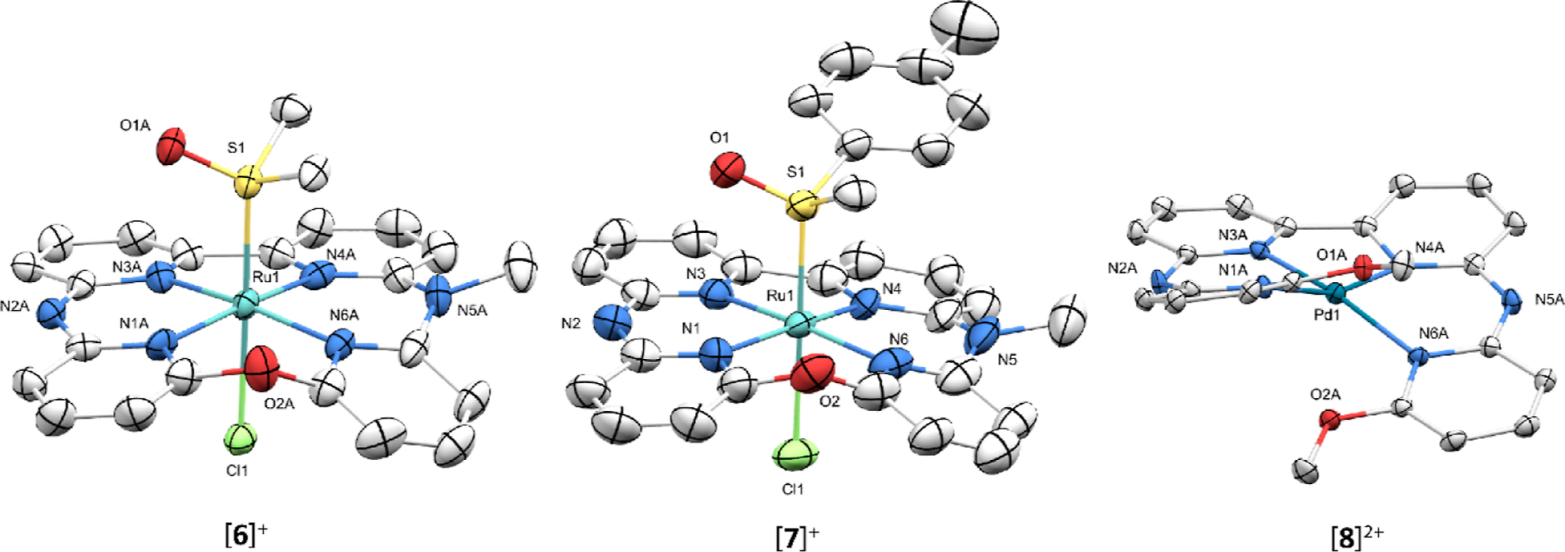
Displacement ellipsoid plots (50% probability level) of the crystal structures of [**6**]OTf, [**7**](OTf)·MeOH, and [**8**](OTf)_2_ at 110(2) K. Counterions, hydrogens, and lattice solvent molecules have been omitted for clarity.

**Scheme 1:**
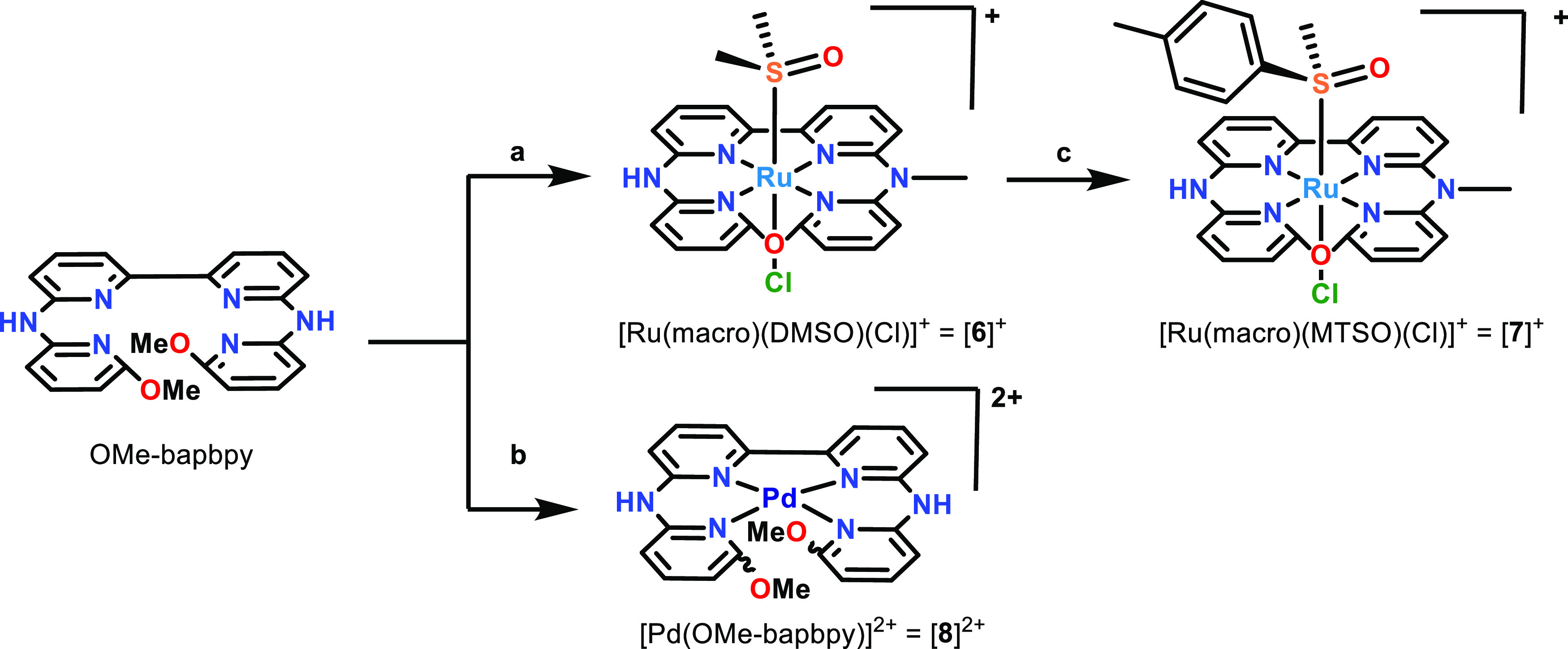
Synthetic Route Toward Compounds [**6**]^+^, [**7**]^+^, and [**8**]^2+^ (a) [Ru(DMSO)_4_(Cl)_2_], EtOH, 85 °C. (b) [Pd(1,5-cyclooctadiene)Cl_2_], EtOH, 85 °C. (c) MTSO, MeOH, 75 °C.

Attempts to prepare a helical compound with increased steric strain generated by methoxy groups in ortho-position to the N atoms of the terminal pyridyl groups of bapbpy produced unexpected results. When reacting OMe-bapbpy with [Ru(DMSO)_4_Cl_2_] under the same conditions as those used to make [**1**]Cl and [**3**]Cl, a solid was obtained ([**6**]Cl) that could initially not be identified by ^1^H NMR. Single crystals suitable for X-ray structure determination were obtained, which allowed identification of the product (Figure 6). According to this crystal structure, the product resulted not only from ligand coordination but also from a subsequent ring-closing reaction, which was accompanied by the removal of one of the methoxy groups, and the methylation of one of the bridging amines. In the X-ray structure of [**6**]OTf, the resulting dissymmetric macrocycle (macro) ligand was coordinated to ruthenium, generating four symmetry-unequivalent pyridyl rings (Scheme 1). This structure was further confirmed by NMR and high-resolution mass spectroscopy (HRMS) ([M + MeCN–2Cl–H]^+^ calcd *m*/*z* = 588.07510, found *m*/*z* = 588.07561) analyses of the product (see full characterization in the Experimental Part). This reaction was robust and reproducible, affording the macrocycle in high yield (∼75%). We hypothesize the following mechanism for the formation of the macrocycle in [**6**]^+^. The close proximity of the two facing methoxy groups arising from the coordination of the OMe-bapbpy ligand to ruthenium(II) might facilitate an intramolecular nucleophilic aromatic substitution via an oxonium ion intermediate, which would subsequently transfer a methyl group, probably in a bimolecular reaction, to the facing amine bridge, thereby forming the final dissymmetric macrocycle.

The dissymmetric nature of the macrocycle in [**6**]^+^ also caused the ruthenium complex to be chiral-at-metal as the metal center was bound in this structure to six non-equivalent heteroatoms. In the crystal structure of [**6**]OTf, both enantiomers were found as the structure was centrosymmetric. When the chiral sulfoxide MTSO ligand was coordinated to this scaffold, to afford [**7**]Cl, the number of peaks in the ^1^H NMR spectrum of [**7**]Cl roughly doubled, which is consistent with the dissymmetric nature of the ring and the formation of a 1:1 mixture of diastereoisomers. Although we were unable to separate these diastereoisomers, we did obtain a crystal structure of [**7**](OTf)(MeOH) (Figure 6), which also showed the presence of both diastereoisomers in the crystal packing. As a note, dissymmetric macrocyclic ligands have been reported in particular for preparing chiral catenanes and other topologically non-obvious mechanically interlocked molecules,^37,38^ but they are often challenging to make. Here, the macrocycle was obtained in a straightforward manner and in good yields, although the presence of the coordinated ruthenium center prevents threading of any molecular building block into the ring.

Finally, to further investigate the role of the metal in this unexpected cyclization reaction, we investigated the coordination of the OMe-bapbpy ligand to the palladium(II) precursor dichloro(1,5-cyclooctadiene)platinum(II) using otherwise identical reaction conditions. Palladium(II) is a d^8^ metal center usually affording square-planar complexes deprived of axial ligands. Surprisingly, this reaction resulted in the simple coordination of the tetrapyridyl ligand to the complex without ring closure, to afford complex [**8**]^2+^. The identity of complex [**8**]^2+^ was supported by NMR, mass spectrometry(ES-MS [Pd(OMe-bapbpy)-H^+^]^+^ calcd *m*/*z* = 505.0, found *m*/*z* 504.9), and single crystal X-ray crystallography of [**8**](OTf)_2_ (Figure 6), which clearly showed an increase in helical strain caused by the terminal methoxy groups, with a N1N3N4N6 dihedral angle that was twice higher (20.8(9)°) than that found in the bapbpy complex [**3**]^+^. Even though macrocycle formation did not occur with palladium(II), this crystal structure also demonstrated the close spatial proximity between the two methoxy groups upon binding of the four pyridyl groups to the metal, with a O2–C1 distance between the “top” oxygen O1 and “bottom” oxygen O2 of 3.01(7) Å. To see if the axial ligands of the ruthenium(II) precursor were pivotal in the macrocycle formation, we also reacted the OMe-bapbpy ligand with a Rh(III) precursor (RhCl_3_ 3H_2_O) under otherwise identical conditions. Here as well, ^1^H NMR and electrospray ionization mass spectrometry (ESI-MS) of the product showed simple coordination of the ligand to rhodium(III) (ES-MS [Rh(OMe-bapbpy)(Cl)_2_]^+^ calc *m*/*z* = 573.0, found *m*/*z* = 572.9), without formation of any macrocycle. This result indicated that the presence of the two axial ligands on ruthenium(II) was not the only reason for the facilitated macrocycle formation in [**6**]^+^, and that the macrocyclization reaction was specific to the chemical reactivity of ruthenium(II).

## Conclusions

In this work, we report the synthesis of helical ruthenium compounds with various levels of steric strain ortho to the terminal pyridyl rings of bapbpy-like ligands. The coordination of an enantiomerically pure (*R*)-sulfoxide axial ligand confirmed that the terminal pyridines of [**3**]^+^ can freely interconvert at room temperature. Variable-temperature NMR allowed the determination of coalescence temperature and energy for both complexes [**1**]^+^ and [**3**]^+^ (43 and 44 kJ/mol, respectively). In [**2**]^+^, the terminal quinolines cannot exchange at room temperature, and heating up to 376 K did not allow to overcome such steric clash. Coordination of the chiral sulfoxide ligand MTSO led to the formation of one major and one minor species, but they could not be separated on HPLC, essentially due to the labile character of the *trans*-chloride ligand. Finally, we report the serendipitous discovery of a robust macrocycle-forming reaction when the methoxy-functionalized OMebapbpy ligand was reacted with ruthenium(II). This reactivity is specific to ruthenium(II) as palladium(II) and rhodium(III) precursors did not result in macrocycle formation but instead led to the simple coordination of the OMebapbpy ligand.

## Experimental part

### Synthesis

All commercially available reagents were ordered from Sigma-Aldrich and were used as received. Bapbpy,^32^ biqbpy,^40^ (*R*)-methyl *p*-tolyl sulfoxide (MTSO),^41^ and compound [**1**]Cl^33^ were prepared according to literature procedures. All reactions were carried out under a N_2_ atmosphere. Filters used were Whatman regenerated cellulose membrane filters, RC60 membrane circles, diam. 47 mm, pore size 1 μm. NMR spectra were recorded on a Bruker, AV-500 spectrometer. HPLC purifications were attempted on a nonchiral column Jupiter 4u Protea 90A, ASXIA and a chiral CYCLOBOND I 2000 DMP column. ESI-MS spectra were recorded by using a MSQ Plus spectrometer in the positive ionization mode. HRMS spectra were recorded on a Waters XEVO-G2 XSQ-TOF mass spectrometer equipped with an electrospray ion source in the positive mode (source voltage 3.0 kV, desolvation gas flow 900 L/h, temperature 250 °C) with resolution *R* = 22,000 (mass range *m*/*z* = 50–2000) and 200 pg/uL Leu-enkephalin (*m*/*z* = 556.2771) as a “lock mass”.

### OMe-bapbpy

6,6′-Dibromo-2,2′-bipyridine (3.0 g, 10 mmol), Pd(dba)2 (295 mg, 0.51 mmol), (Rac)-BINAP (500 mg, 0.80 mmol), and potassium *t*-butoxide (4.2 g, 38 mmol) were added to a flask containing toluene (300 mL). Afterward, 2-amino-6-methoxypyridine (3.1 mL, 29 mmol) was added, and the reaction was refluxed overnight at 110 °C. The next day, the reaction was allowed to cool down to room temperature, after which water (150 mL) was added. The mixture was stirred for 1 h, after which it was filtered, and the solid washed with water (2 × 25 mL). The solid residue was finally dried under vacuum to afford the title compound as a beige-colored solid. Yield: 3.6 g, 8.9 mmol, 90%. ^1^H NMR (500 MHz, DMSO): δ 9.66 (s, 2H), 7.89–7.80 (m, 4H), 7.75 (d, *J* = 6.7 Hz, 2H), 7.64 (t, *J* = 7.9 Hz, 2H), 7.49 (d, *J* = 7.9 Hz, 2H), 6.31 (d, *J* = 7.9 Hz, 2H), 3.89 (s, 6H). ^13^C NMR (126 MHz, DMSO): δ 162.36 (Cq), 153.69 (Cq), 153.53 (Cq), 152.49 (Cq), 140.39 (CH), 138.33 (CH), 112.43 (CH), 112.08 (CH), 103.10 (CH), 100.70 (CH), 53.02 (CH_3_). HRMS [M + H]^+^: 401.17205 (calculated), 401.17204 (measured).

### [**2**]Cl

Biqbpy (90 mg, 0.21 mmol) and [Ru(DMSO)_4_(Cl)_2_] (0.10 g, 0.21 mmol) were dissolved in degassed ethanol (25 mL). The solution was refluxed for 3 days at 80 °C under a N_2_ atmosphere. The mixture was concentrated in vacuo and reprecipitated from MeOH (5 mL) with diethyl ether (50 mL) afford the title compound [**3**]Cl as a dark-brown powder. Yield: 0.15 g, 0.20 mol, 96%. ^1^H NMR (500 MHz, DMSO): δ 12.31 (s, 1H), 12.08 (s, 1H), 8.55 (d, *J* = 6.9 Hz, 1H), 8.49 (d, *J* = 6.9 Hz, 1H), 8.36 (d, *J* = 8.8 Hz, 1H), 8.30 (d, *J* = 8.8 Hz, 1H), 8.25 (t, *J* = 8.0 Hz, 1H), 8.18 (t, *J* = 8.0 Hz, 1H), 8.12 (d, *J* = 8.8 Hz, 1H), 8.05 (d, *J* = 8.3 Hz, 1H), 7.99 (d, *J* = 8.9 Hz, 1H), 7.90 (d, *J* = 7.2 Hz, 1H), 7.72 (d, *J* = 8.8 Hz, 1H), 7.68 (dd, *J* = 7.9, 1.6 Hz, 1H), 7.59 (dd, *J* = 7.8, 1.7 Hz, 1H), 7.52 (d, *J* = 8.8 Hz, 1H), 6.96 (t, *J* = 7.4 Hz, 1H), 6.88 (t, *J* = 6.8 Hz, 1H), 6.36 (ddd, *J* = 8.6, 6.9, 1.6 Hz, 1H), 6.29 (ddd, *J* = 8.8, 6.9, 1.6 Hz, 1H), 2.54 (s, 6H). ^13^C NMR (126 MHz, DMSO): δ 156.91 (Cq), 155.85 (Cq), 154.37 (Cq), 153.01 (Cq), 151.24 (Cq), 150.66 (Cq), 148.55 (Cq), 148.37 (Cq), 138.78 (CH), 138.52 (CH), 137.96 (CH), 137.02 (CH), 130.55 (CH), 129.11 (CH), 128.01 (CH), 127.68 (CH), 126.72 (CH), 126.63 (CH), 124.78 (Cq), 124.51 (Cq), 124.37 (CH), 123.96 (CH), 118.08 (CH), 117.55 (CH), 116.07 (CH), 115.90 (CH), 115.00 (CH), 113.87 (CH), 40.36 (CH_3_). HRMS [M + MeCN–2Cl–DMSO]^2+^: 291.55277 (calculated), 291.55249 (measured). Elem. Anal. Calcd. for [C_30_H_28_Cl_2_N_6_O_2_RuS] + H_2_O: C, 50.85; H, 3.89; N, 11.86. Found: C, 50.72; H, 3.73; N, 11.69.

### [**3**]Cl

[**1**]Cl (30 mg, 0.051 mmol) and MTSO (0.21 g, 1.3 mmol) were added to degassed methanol (10 mL). The solution was refluxed overnight, after which the solution was allowed to cool to room temperature and precipitated by addition of diethyl ether (10 mL). The precipitate was filtered, washed with diethyl ether (2 × 50 mL), and dried under vacuum to afford the desired compound as a red-brown-colored solid. Yield: 37 mg, 0.051 mmol, 100%. ^1^H NMR (500 MHz, DMSO): δ 11.66 (s, 1H), 11.13 (s, 1H), 8.44 (dd, *J* = 6.1, 1.7 Hz, 1H), 8.37 (dd, *J* = 6.1, 1.7 Hz, 1H), 8.29 (dd, *J* = 7.9, 1.0 Hz, 1H), 8.18–8.06 (m, 2H), 7.95 (ddd, *J* = 8.6, 7.1, 1.7 Hz, 1H), 7.89 (t, *J* = 8.0 Hz, 1H), 7.80 (ddd, *J* = 8.6, 7.0, 1.7 Hz, 1H), 7.69 (d, *J* = 7.3 Hz, 1H), 7.58 (dd, *J* = 8.5, 1.4 Hz, 1H), 7.29–7.22 (m, 2H), 7.13 (ddd, *J* = 7.3, 6.1, 1.4 Hz, 1H), 7.04 (ddd, *J* = 7.3, 6.0, 1.4 Hz, 1H), 6.87 (d, *J* = 8.0 Hz, 2H), 6.42 (d, *J* = 8.3 Hz, 2H), 2.43 (s, 3H), 2.21 (s, 3H). ^13^C NMR (126 MHz, DMSO): δ 155.67 (Cq), 155.22 (Cq), 153.06 (CH), 153.01 (CH), 152.66 (Cq), 152.25 (Cq), 151.12 (Cq), 150.37 (Cq), 140.59 (Cq), 140.34 (Cq), 138.01 (CH), 137.61 (CH), 137.47 (CH), 136.83 (CH), 128.69 (CH), 122.71 (CH), 117.13 (CH), 117.01 (CH), 116.79 (CH), 114.57 (CH), 114.40 (CH), 114.11 (CH), 114.03 (CH), 43.78 (CH_3_), 20.66 (CH_3_). HRMS [M + MeCN–2Cl–H]^+^: 636.11250 (calculated), 636.11207 (measured). Elem. Anal. Calcd. For [C_28_H_28_Cl_2_N_6_O_2_RuS] + H_2_O: C, 49.12; H, 4.12; N, 12.28. Found: C, 49.13; H, 4.13; N, 12.27.

### [**4**]Cl

[**2**]Cl (42 mg, 0.08 mmol) and MTSO (0.17 g, 1.1 mmol) were added to degassed methanol (15 mL). The solution was refluxed for 3 days at 75 °C under a N_2_ atmosphere. After concentration in vacuo, the solid residue was sonicated in ethyl acetate (15 mL) and filtered and washed with diethyl ether (2 × 50 mL) to obtain the title compound [**4**]Cl as a dark-brown powder. Yield: 45 mg 0.06, 75%. HRMS [M – 2Cl–H]^+^: 695.11704 (calculated) 695.11677 (measured). Elem. Anal. Calcd. for [C_36_H_30_Cl_2_N_6_ORuS]: C, 56.40; H, 3.94; N, 10.96. Found: C, 56.28; H, 3.93; N, 10.94.

### [**5**](PF_6_)_2_

[**2**]Cl (53 mg, 0.08 mmol) and (*R*)-4-(1-hydroxyethyl)pyridine (0.45 g, 3.6 mmol) were added to a flask containing deoxygenated demineralized water (45 mL). The solution was stirred for 1 day at 80 °C under a N_2_ atmosphere. The suspension was filtered, and saturated aqueous KPF_6_ solution (5 mL) was added to the filtrate, after which a precipitate formed. This precipitate was filtered, washed with water (2 × 20 mL), and dried under vacuo to obtain the compound as dark-brown/red solid. Yield: 0.68 g, 0.07 90%. ^1^H NMR (500 MHz, acetone): δ 8.52–8.39 (m, 3H), 8.34 (dd, *J* = 7.9, 0.9 Hz, 1H), 8.19 (t, *J* = 8.0 Hz, 1H), 7.90 (d, *J* = 8.3 Hz, 1H), 7.80 (d, *J* = 8.8 Hz, 1H), 7.68 (dd, *J* = 7.9, 1.6 Hz, 1H), 7.15 (d, *J* = 8.9 Hz, 1H), 7.11 (d, *J* = 6.2 Hz, 1H), 6.91 (t, *J* = 7.4 Hz, 1H), 6.38–6.30 (m, 1H), 4.61 (d, *J* = 6.8 Hz, 1H), 4.44 (s, 1H), 1.10 (dd, *J* = 6.5, 4.1 Hz, 3H). ^13^C NMR (126 MHz, acetone): δ 156.77 (Cq), 155.49 (Cq), 153.76 (CH), 152.49 (Cq), 149.83 (Cq), 147.82 (Cq), 138.46 (CH), 136.43 (CH), 127.54 (CH), 127.24 (CH), 126.95 (CH), 124.48 (Cq), 123.94 (CH), 121.56 (CH), 117.44 (CH), 115.68 (CH), 115.48 (CH), 65.73, 65.68, 23.08 (CH_3_). HR-MS [M – 2(PF_6_)]^2+^: 394.10808 (calculated), 394.10743 (measured). Elem. Anal. Calcd. for [C_42_H_38_F_12_N_8_O_2_P_2_Ru] + 0.2 KPF_6_: C, 45.26; H, 3.44; N, 10.05. Found: C, 45.54; H, 3.31; N, 9.36.

### [**6**]Cl

The ligand OMe-bapbpy (0.16 g, 0.41 mmol) and [Ru(DMSO)_4_(Cl)_2_] (0.20 g, 0.41 mmol) were added to a flask containing deoxygenated EtOH (25 mL). The solution was refluxed over the weekend at 85 °C under a N_2_ atmosphere. Afterward, the mixture was concentrated in vacuo and re-precipitated from MeOH (10 mL) and an excess of diethyl ether (50 mL), filtered and washed with diethyl ether (2 × 50 mL), and dried overnight in vacuo to obtain [**6**]Cl as an orange-red solid. Yield: 0.21 g, 0.37, 84%.

^1^H NMR (500 MHz, DMSO): δ 11.92 (s, 1H), 8.46 (d, *J* = 7.2 Hz, 1H), 8.37 (d, *J* = 7.0 Hz, 1H), 8.22 (dd, *J* = 8.4, 7.8 Hz, 1H), 8.12 (dt, *J* = 9.3, 8.2 Hz, 2H), 7.98 (t, *J* = 8.0 Hz, 1H), 7.69 (d, *J* = 7.5 Hz, 1H), 7.64 (d, *J* = 7.6 Hz, 1H), 7.39–7.32 (m, 2H), 7.14 (d, *J* = 7.0 Hz, 1H), 6.93 (d, *J* = 6.8 Hz, 1H), 3.75 (s, 3H), 2.53 (s, 3H), 2.40 (s, 3H). ^13^C NMR (126 MHz, DMSO): δ 156.46 (Cq), 156.15 (Cq), 155.07 (Cq), 154.82 (Cq), 154.26 (Cq), 154.07 (Cq), 149.29 (Cq), 148.69 (Cq), 140.86 (CH), 140.07 (CH), 137.69 (CH), 137.27 (CH), 116.90 (CH), 116.54 (CH), 115.56 (CH), 114.19 (CH), 112.31 (CH), 109.03 (CH), 108.22 (CH), 104.74 (CH), 44.55 (CH3), 43.83 (CH3), 43.31 (CH3). HRMS [M + MeCN–2Cl–H]^+^: 588.07510 (calculated), 588.07561 (measured). Elem. Anal. Calcd. for [C_23_H_22_Cl_2_N_6_O_2_RuS] + 1.4 H_2_O: C, 42.91; H, 3.88; N, 13.06. Found: C, 42.88; H, 3.64; N, 12.96.

### [**7**]Cl

[**6**]Cl (0.11 g, 0.17 mmol) and MTSO (0.54 g, 3.5 mmol) were added to degassed methanol (25 mL). The solution was refluxed for 3 days at 75 °C under a N_2_ atmosphere. The reaction mixture was concentrated in vacuo, and ethyl acetate (10 mL) was added. The suspension was then sonicated for 20 min at room temperature in a Brandson 3510 ultrasonic cleaner. The suspension was filtered, and the solid fraction was dried overnight in vacuo to obtain the title compound as yellow solid. Yield: 0.10 mg, 0.15 mmol, 87%. ^1^H NMR (500 MHz, DMSO): δ 8.40 (d, *J* = 7.9 Hz, 1H), 8.37–8.29 (m, 2H), 8.23 (d, *J* = 7.7 Hz, 1H), 8.19–8.01 (m, 6H), 7.98 (t, *J* = 8.0 Hz, 1H), 7.92 (t, *J* = 8.0 Hz, 1H), 7.59–7.45 (m, 5H), 7.39 (d, *J* = 7.9 Hz, 1H), 7.33 (d, *J* = 8.3 Hz, 1H), 7.26 (t, *J* = 9.1 Hz, 2H), 7.12 (d, *J* = 8.0 Hz, 1H), 7.11–6.98 (m, 6H), 6.93 (d, *J* = 7.9 Hz, 1H), 6.81 (d, *J* = 7.8 Hz, 1H), 6.64 (d, *J* = 8.0 Hz, 2H), 6.58 (d, *J* = 8.0 Hz, 2H), 3.63 (s, 3H), 3.54 (s, 3H), 2.90 (s, 3H), 2.78 (s, 3H), 2.70 (s, 2H), 2.54 (s, 1H), 2.37 (s, 2H), 2.24 (d, *J* = 2.5 Hz, 6H). HR-MS [M – Cl]^+^: 664.10707 (calculated), 664.10640 (measured). Elem. Anal. Calcd. for [C_29_H_26_Cl_2_N_6_O_2_RuS] + 0.5 H_2_O: C, 49.50; H, 3.87; N, 11.94. Found: C, 48.93; H, 3.71; N, 12.17.

### [**8**](Cl)_2_

The ligand OMe-bapbpy (71 mg, 0.18 mmol) and [Pd(1,5-cyclooctadiene)(Cl)_2_] (50 mg, 0.18 mmol) were added to a one-necked round-bottom flask containing deoxygenated EtOH (25 mL). The solution was refluxed over the weekend at 85 °C under a N_2_ atmosphere. To ensure that no side products were removed, the reaction mixture was concentrated in vacuo and afforded in [**8**](Cl)_2_ in quantitative yield. ^1^H NMR (500 MHz, DMSO): δ 12.98 (s, 1H), 8.28 (dd, *J* = 8.5, 7.6 Hz, 1H), 8.18 (d, *J* = 6.6 Hz, 1H), 8.13 (t, *J* = 8.1 Hz, 1H), 7.88 (d, *J* = 7.4 Hz, 1H), 7.45 (d, *J* = 7.2 Hz, 1H), 6.72 (d, *J* = 7.3 Hz, 1H), 3.41 (s, 3H). ^13^C NMR (126 MHz, DMSO): δ 163.17 (Cq), 154.24 (Cq), 146.79 (Cq), 146.13 (Cq), 143.88 (CH), 141.64 (CH), 117.22 (CH), 116.12 (CH), 107.01 (CH), 98.87 (CH), 56.62 (CH_3_). ES-MS [M – 2Cl–H]^+^: 505.0 (calculated) 504.9 (measured).

### [**9**](Cl)

The ligand OMe-bapbpy (96 mg, 0.24 mmol) and [Rh(Cl)_3_]3H_2_O (50 mg, 0.19 mmol) were added to one-necked round-bottom flask containing deoxygenated EtOH (25 mL). The solution was refluxed over the weekend at 85 °C under a N_2_ atmosphere. To ensure that no side products were removed, the reaction mixture was concentrated in vacuo and was analyzed without further purification and afforded [Rh(OMe-bapbpy)(Cl)_2_]Cl in quantitative yield. ^1^H NMR (500 MHz, DMSO): δ 11.80 (s, 1H), 8.31 (d, *J* = 7.6 Hz, 1H), 8.20 (t, *J* = 8.0 Hz, 1H), 8.03 (t, *J* = 8.1 Hz, 1H), 7.59 (d, *J* = 8.4 Hz, 1H), 7.18 (d, *J* = 8.0 Hz, 1H), 6.65 (d, *J* = 8.2 Hz, 1H), 3.40 (s, 3H). ES-MS [M – Cl]^+^: 573.0 (calculated) 572.9 (measured).

## Supporting Information Available

HPLC trace for the separation of [**4**]Cl, NMR and mass characterization spectra of ligand 6-OMebapbpy and compounds [**2**]Cl–[**9**]Cl, and crystallographic data (PDF)
