# Supplementary material for: Ruthenium-Locked Helical Chirality: A Barrier of Inversion and Formation of an Asymmetric Macrocycle
Source: Inorg Chem. 2022 Sep 29;61(40):16045–54. doi: 10.1021/acs.inorgchem.2c02447 (PMC9554910; doi:10.1021/acs.inorgchem.2c02447)
Supplement: Supplementary file 1 — ic2c02447_si_001.pdf [file ic2c02447_si_001.pdf]

Supporting Information

for

Ruthenium-locked helical chirality: barrier of inversion and formation of an asymmetric macrocycle

Corjan van de Griend,[a] Johannes J. van de Vijver,[a] Maxime A. Siegler,[b] Remus T. Dame,[a] Sylvestre Bonnet*[a]

[a] Leiden Institute of Chemistry, Leiden University, Einsteinweg 55, 2333CC Leiden, The Netherlands
[b] Small molecule X-ray facility, Department of Chemistry, John Hopkins University, Baltimore, Maryland 21218, United States

Corresponding author: bonnet@chem.leidenuniv.nl

Contents

1.1 Crystal structures of diastereomers and epimers. 2
1.2 HPLC 3
1.3 Characterization 4
1.3.1 OMe-bapbpy 4
1.3.2 [Ru(biqbpy)(DMSO)(Cl)]Cl [2]Cl 6
1.3.3 [Ru(bapbpy)(MTSO)(Cl)]Cl [3]Cl 9
1.3.4 [Ru(biqbpy)(MTSO)(Cl)]Cl [4]Cl 12
1.3.5 [Ru(biqbpy)(EtOPy)$_2$](PF$_6$)$_2$ [5](PF$_6$)$_2$ 13
1.3.6 [Ru(macro)(DMSO)Cl]Cl [6]Cl 16
1.3.7 [Ru(macro)(MTSO)Cl]Cl [7]Cl 18
1.3.8 [Pd(OMe-babppy)](Cl)$_2$ [8]Cl 20
1.3.9 [Rh(OMe-bapbpy)Cl$_2$](Cl) 22
1.4 Crystal structures 25
1.4.1 [Ru(bapbpy)(MTSO)(Cl)]PF$_6$, [3]PF$_6$ 25
1.4.2 [Ru(biqbpy)(EtOHPy)$_2$](PF$_6$)$_2$, [5](PF$_6$)$_2$ 27
1.4.3 [Ru(macro)(DMSO)(Cl)]OTf, [6]OTf 29
1.4.4 [Ru(macro)(MTSO)(Cl)](OTf)(MeOH), [7](OTf)(MeOH) 31
1.4.5 [Pd(OMe-bapbpy)](OTf)$_2$, [8](OTf)$_2$ 33
2 References 35

1.1 Crystal structures of diastereomers and epimers.

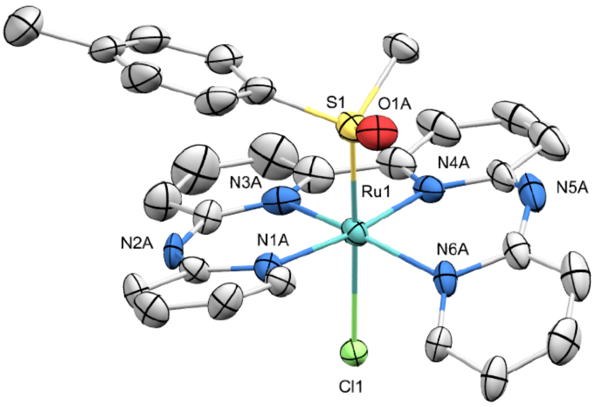

(*P*,*R*)[**3**]$^+$

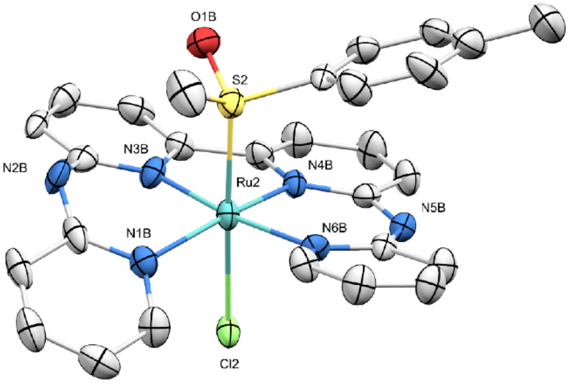

(*M*,*R*)[**3**]$^+$

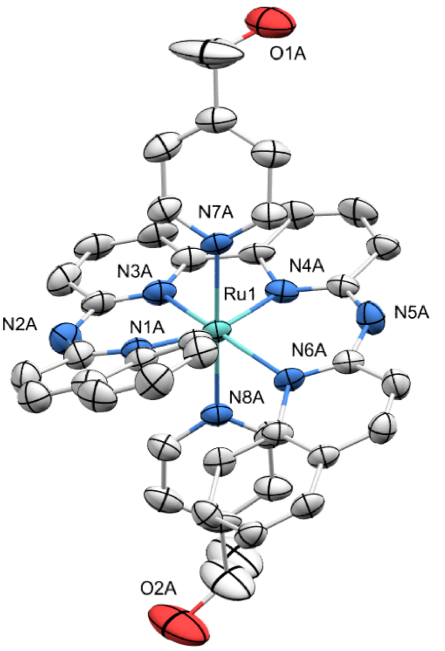

(*P*,*R*,*R*)[**5**]$^{2+}$

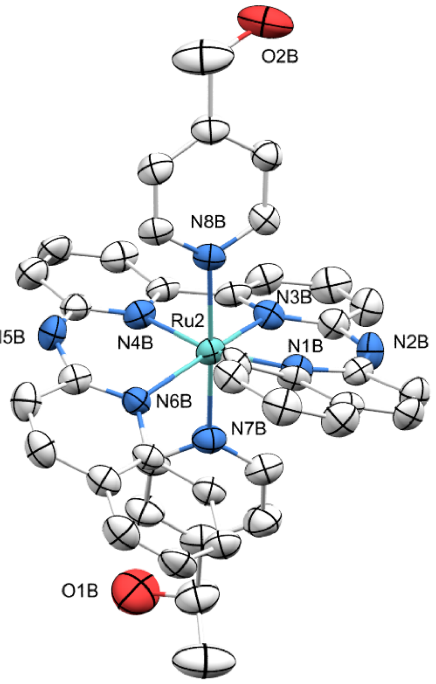

(*M*,*R*,*R*)[**5**]$^{2+}$

Figure S1. Displacement ellipsoid plots (50% probability level) for (*P*,*R*) and (M,R) diastereomers of [**3**]PF_6 and (*P*,*R,R*) and (*M*,*R*,*R*) epimers of [**5**]$(PF_6)_2$ at 110(2) K. Counter ions and hydrogens have been omitted for clarity.

1.2 HPLC

Sample Name:	CG-85	*Inj. Vol.:*	25.0
Sample Type:	unknown	*Dilution Factor:*	1.0000
Program:	CJ-Chiral-prep-seperation-2mllmin	*Operator:*	n.a.
Inj. Date/Time:	16.11.21 15:06	*Run Time:*	20.09

No.	Time min	Peak Name	Type	Area mAU*min	Height mAU	Amount n.a.
		TOTAL:		0.00	0.00	0.00

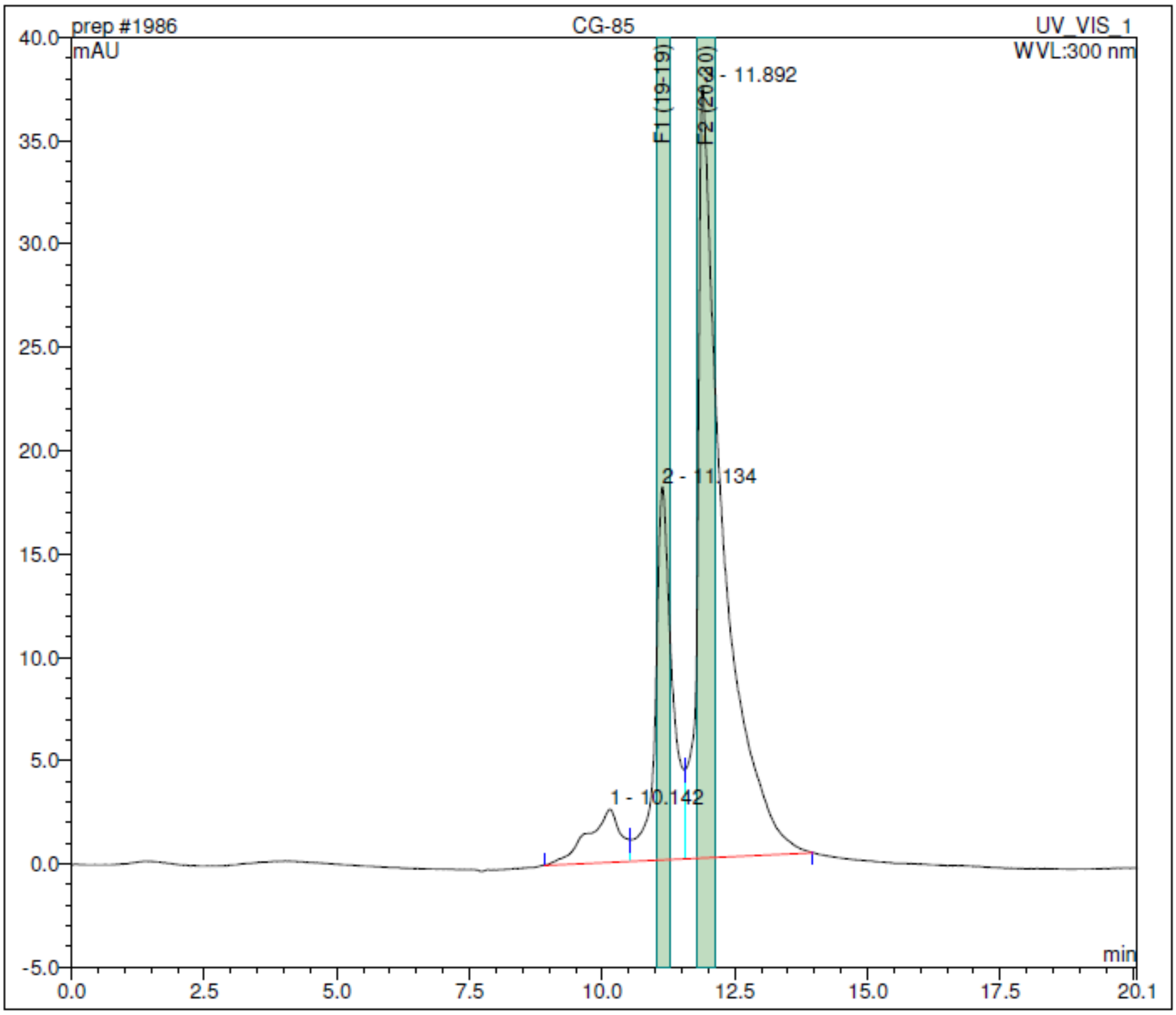

Figure S2. HPLC traces of [**4**]Cl with 0.1 M NH4Cl in MeOH eluent on the cyclobond I 2000 DMP column.

1.3 Characterization

1.3.1 OMe-bapbpy

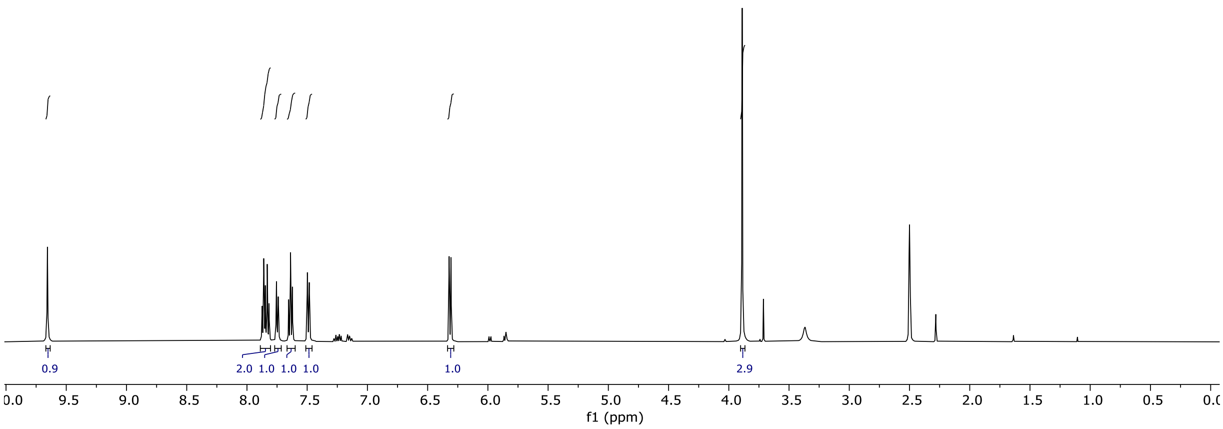

Figure S3. ^{1}H-NMR of OMe-Bapbpy in DMSO-d_6.

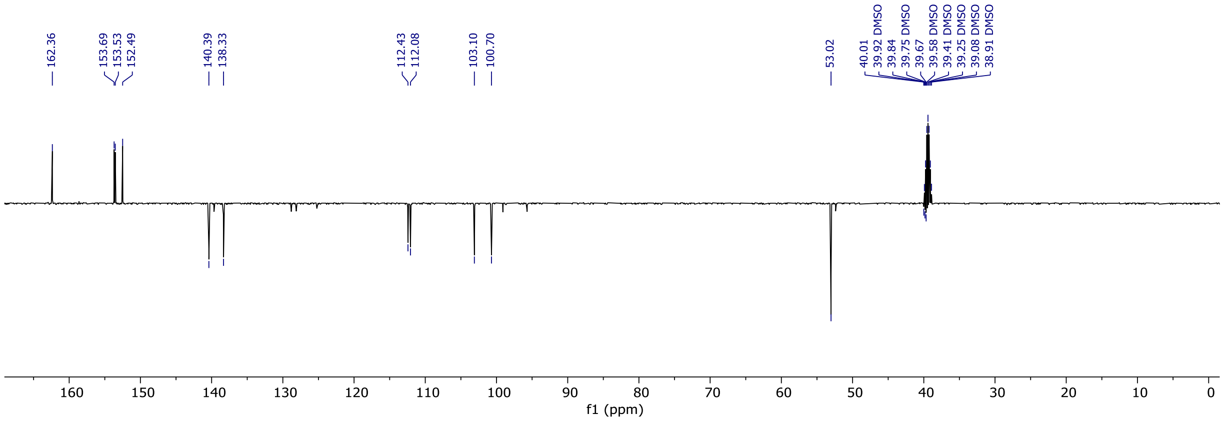

Figure S4. ^{13}C-APT-NMR of OMe-Bapbpy in DMSO-d_6.

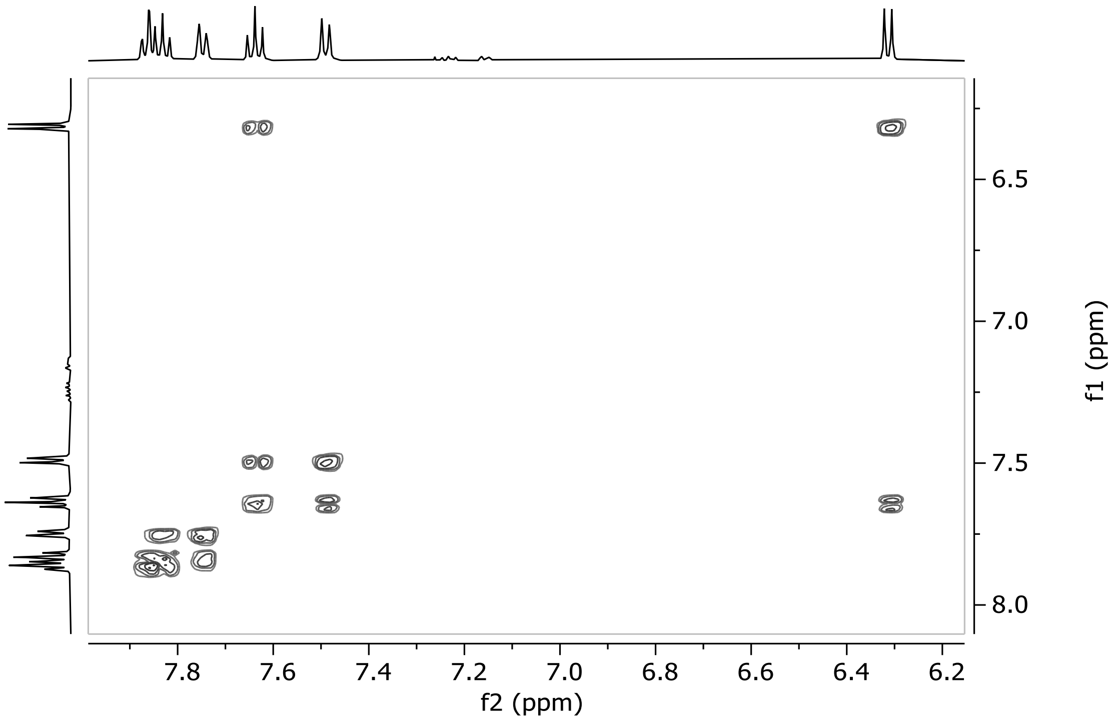

Figure S5 ^{1}H-^{1}H-Cosy-NMR of OMe-bapbpy in DMSO-d_6.

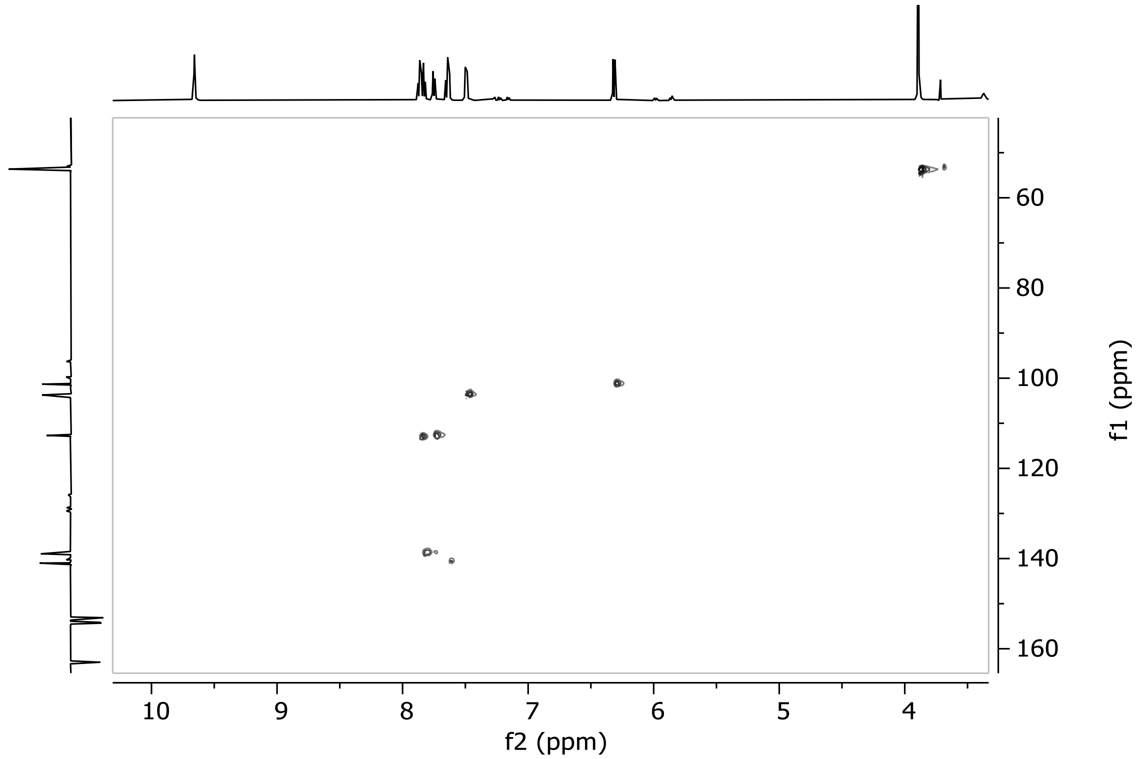

Figure S6. ^{1}H-^{13}C-HSQC of OMe-bapbpy in DMSO-d_6.

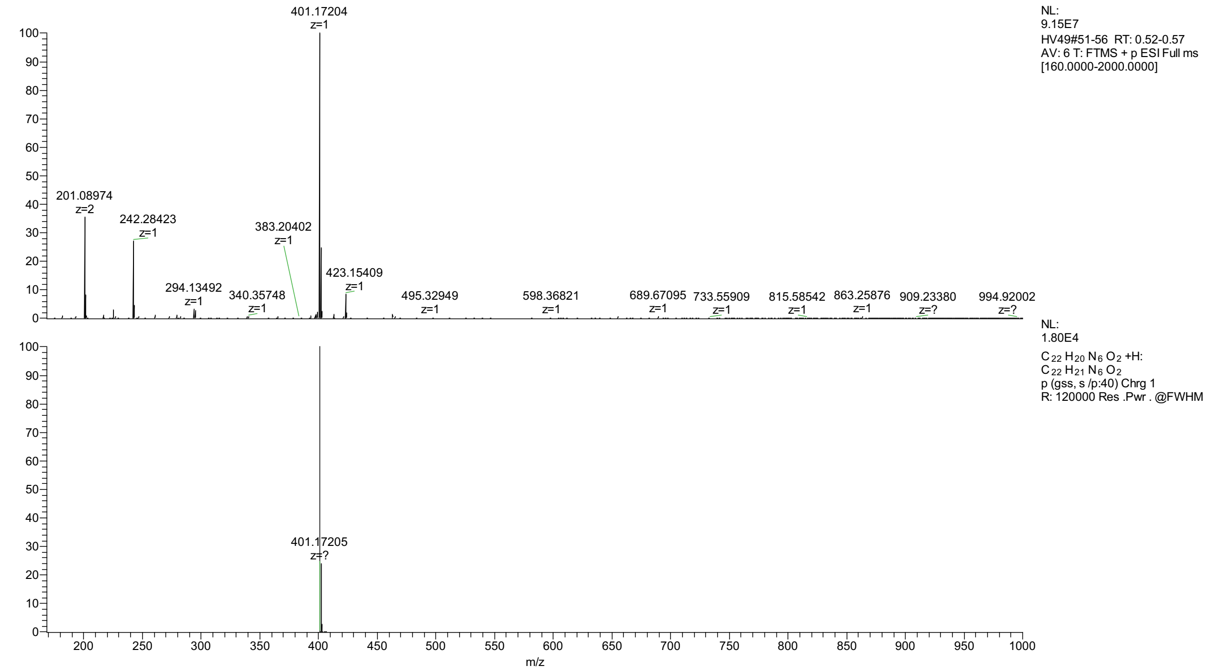

Figure S7. HR-MS of OMe-bapbpy. Top measured, bottom calculated for [M+H]+.

1.3.2 [Ru(biqbpy)(DMSO)(Cl)]Cl [2]Cl

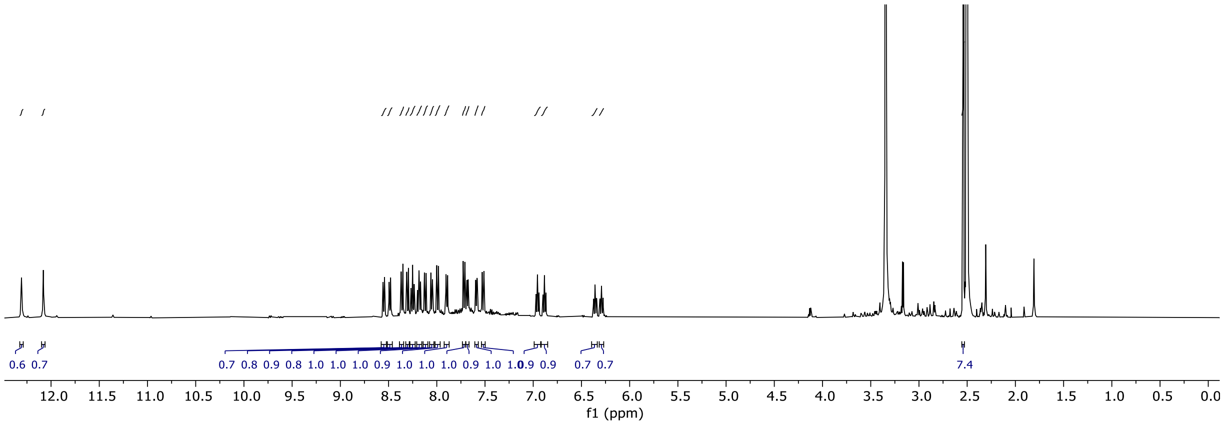

Figure S8. ^{1}H-NMR of [**2**]Cl in DMSO-d_6.

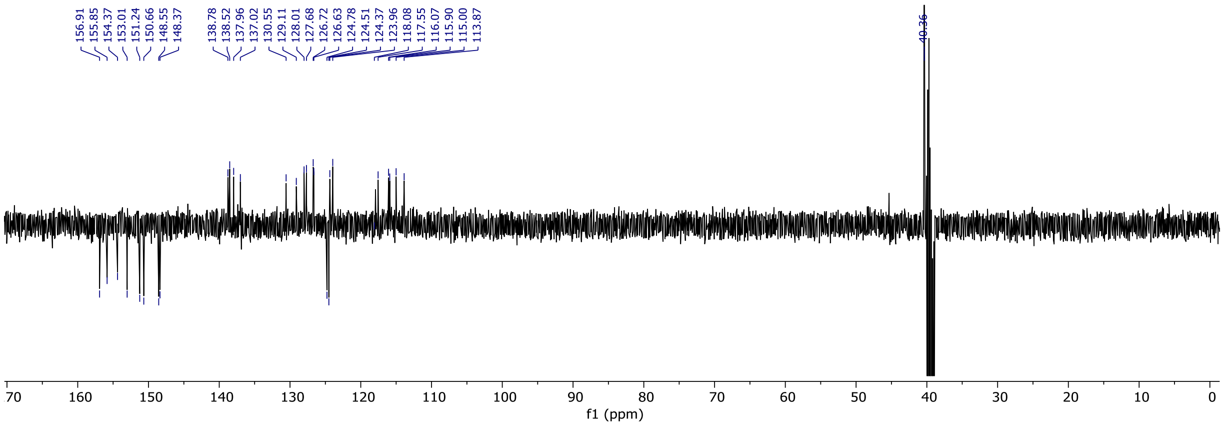

Figure S9. ^{13}C-APT-NMR of [**2**]Cl in DMSO-d_6.

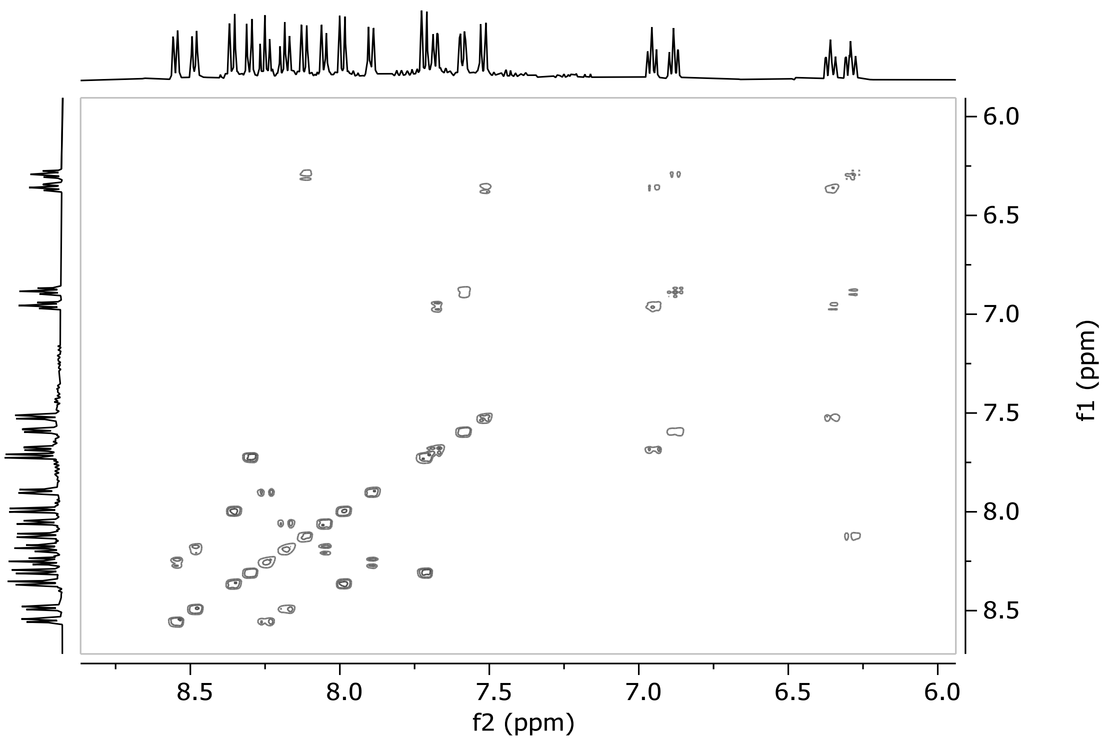

Figure S10. ^{1}H-^{1}H-Cosy-NMR of [**2**]Cl in DMSO-d_6.

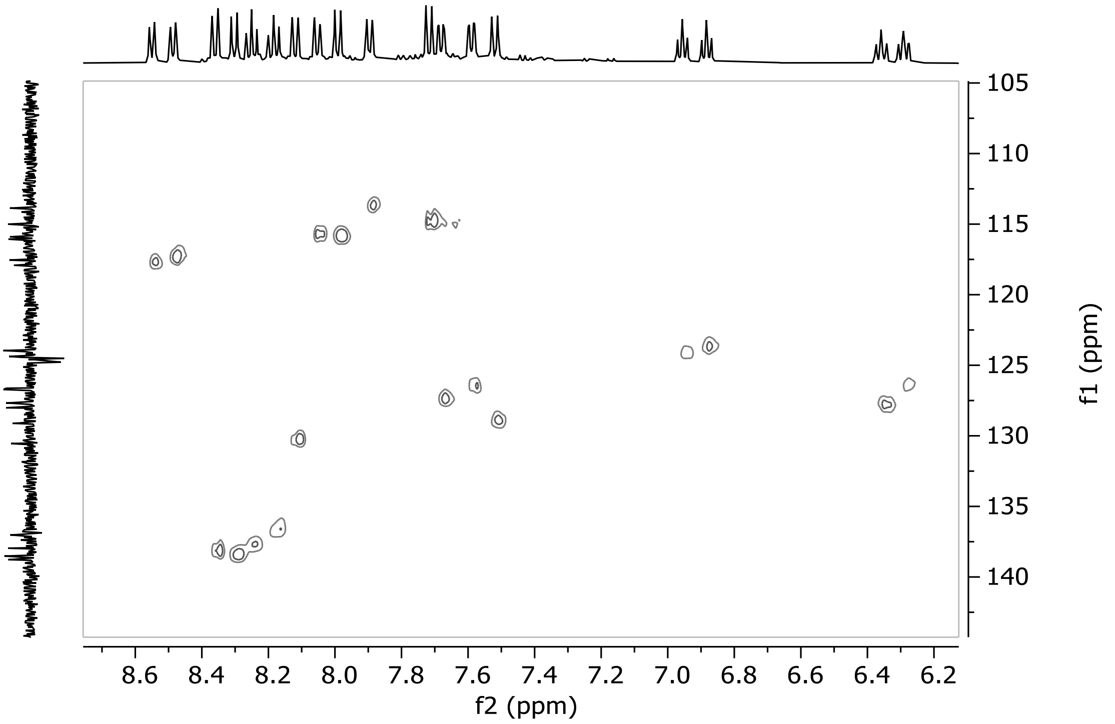

Figure S11. ^{1}H-^{13}CHSQC of [**2**]Cl in DMSO- d_6.

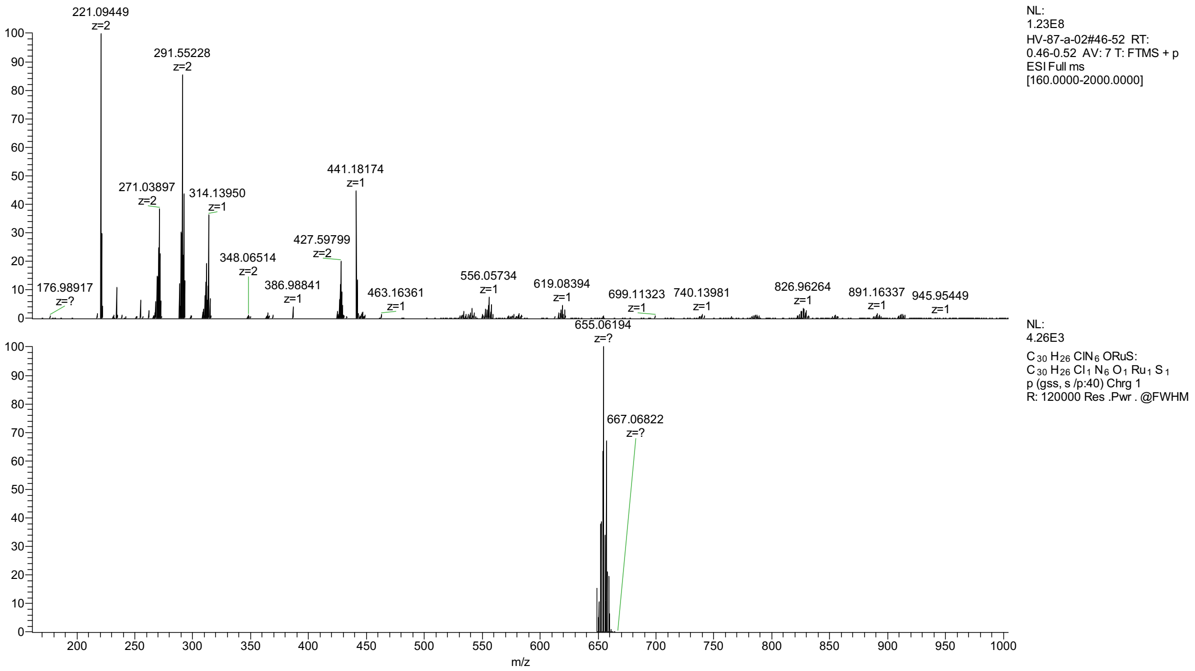

Figure S12. HR-MS of [**2**]Cl. Top measured and bottom calculated for $[M]^{+}$.

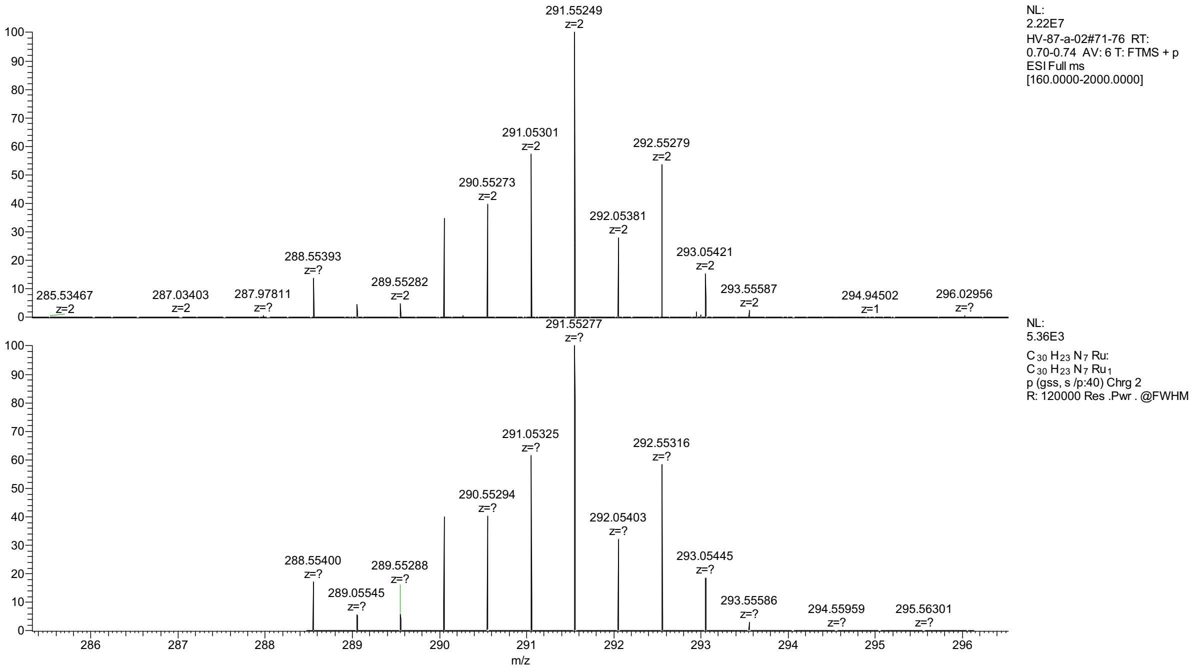

Figure S13. HR-MS of [**2**]Cl. Top measured and bottom calculated for $[M{+}MeCN{-}2Cl{-}DMSO]^{2+}$.

1.3.3 [Ru(bapbpy)(MTSO)(Cl)]Cl [3]Cl

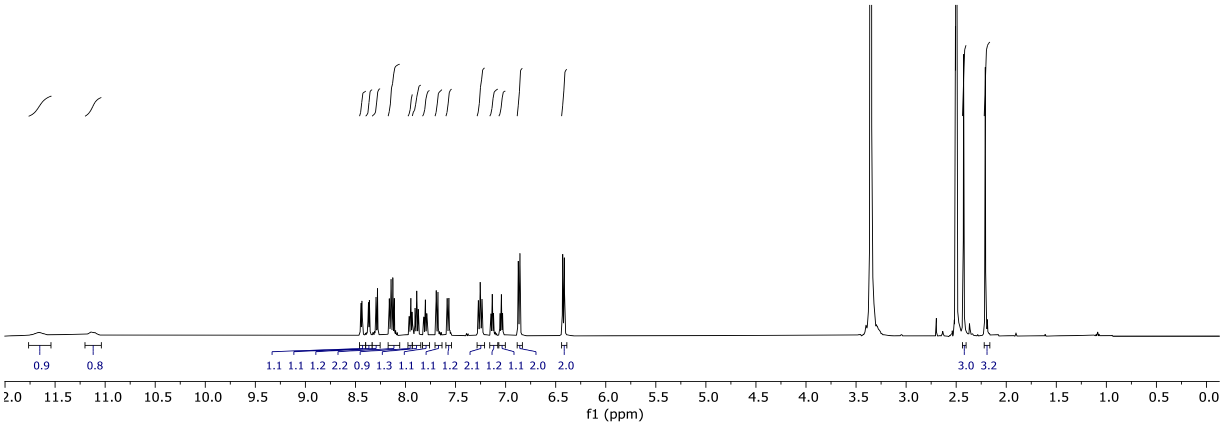

Figure S14. ^{1}H-NMR of [**3**]Cl in DMSO-d_6.

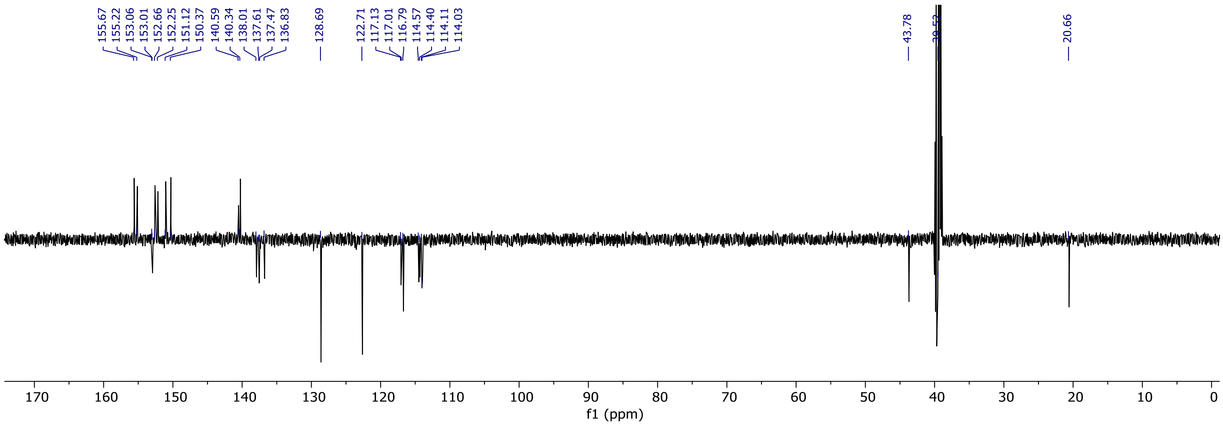

Figure S15. ^{13}C-APT-NMR of [**3**]Cl in DMSO-d_6.

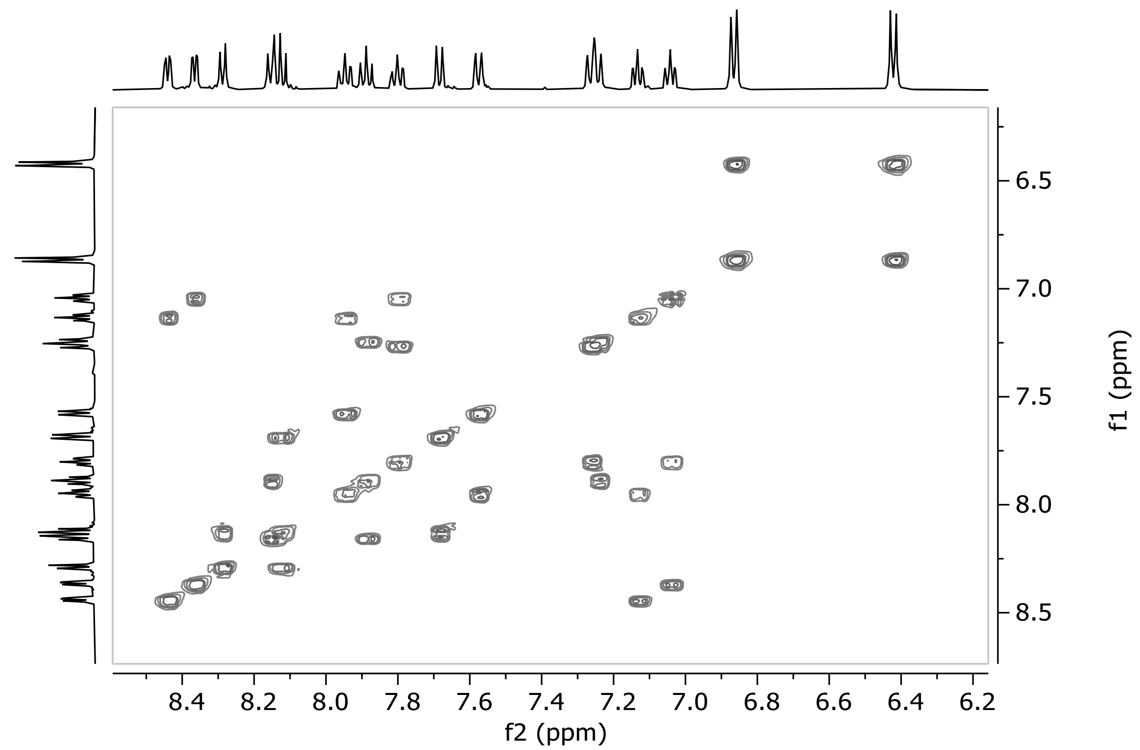

Figure S16. ^{1}H-^{1}H-Cosy-NMR of [**3**]Cl in DMSO-d_6.

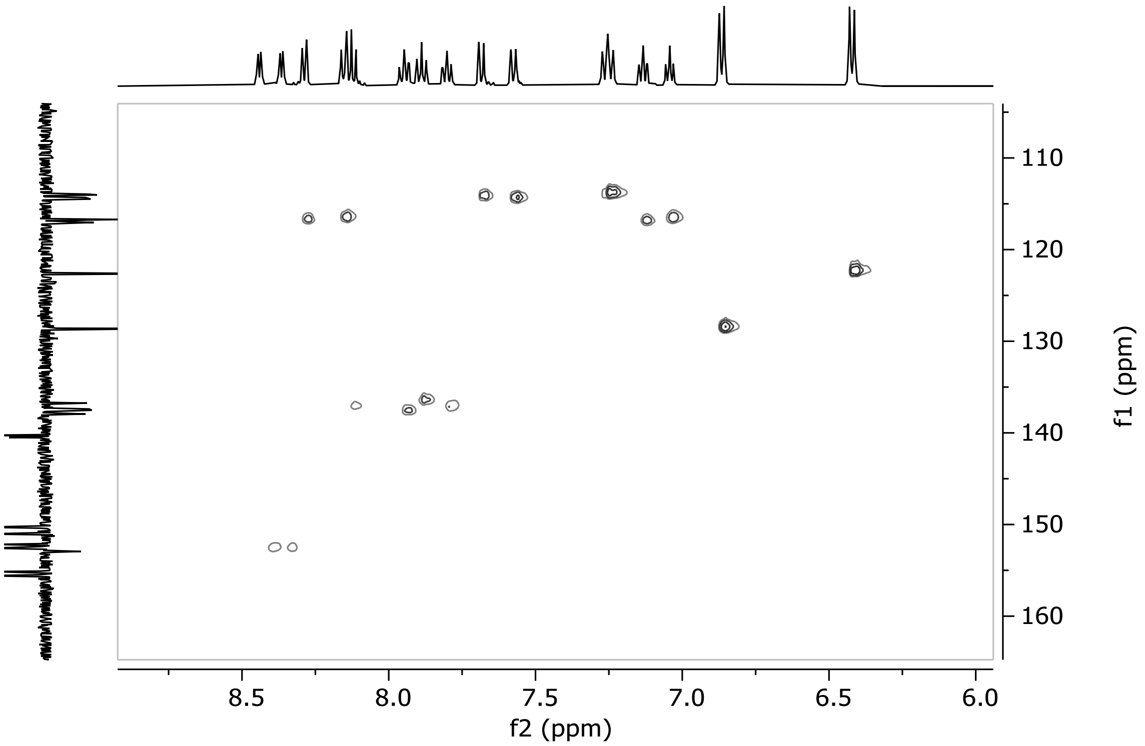

Figure S17. ^{1}H-^{13}C-HSQC of [**3**]Cl in DMSO-d_6.

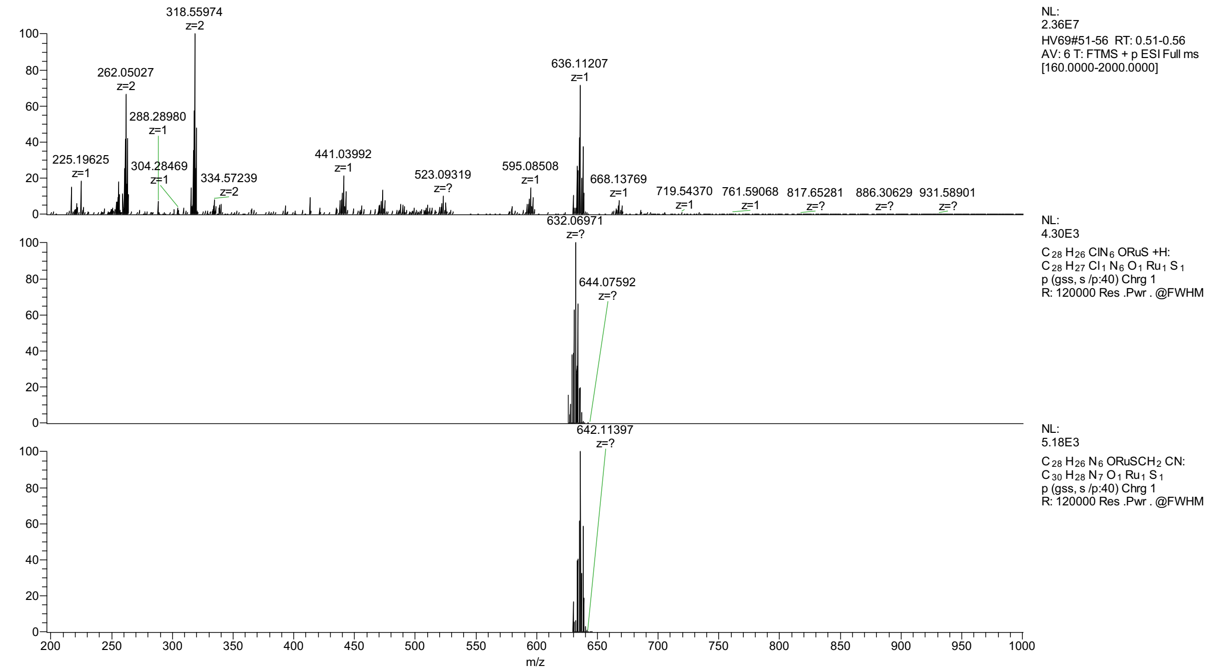

Figure S18. HR-MS of [**3**]Cl. Top measured, middle calculated for $[M+H-Cl]^+$ and bottom calculated for $[M+MeCN–2Cl-H]^+$.

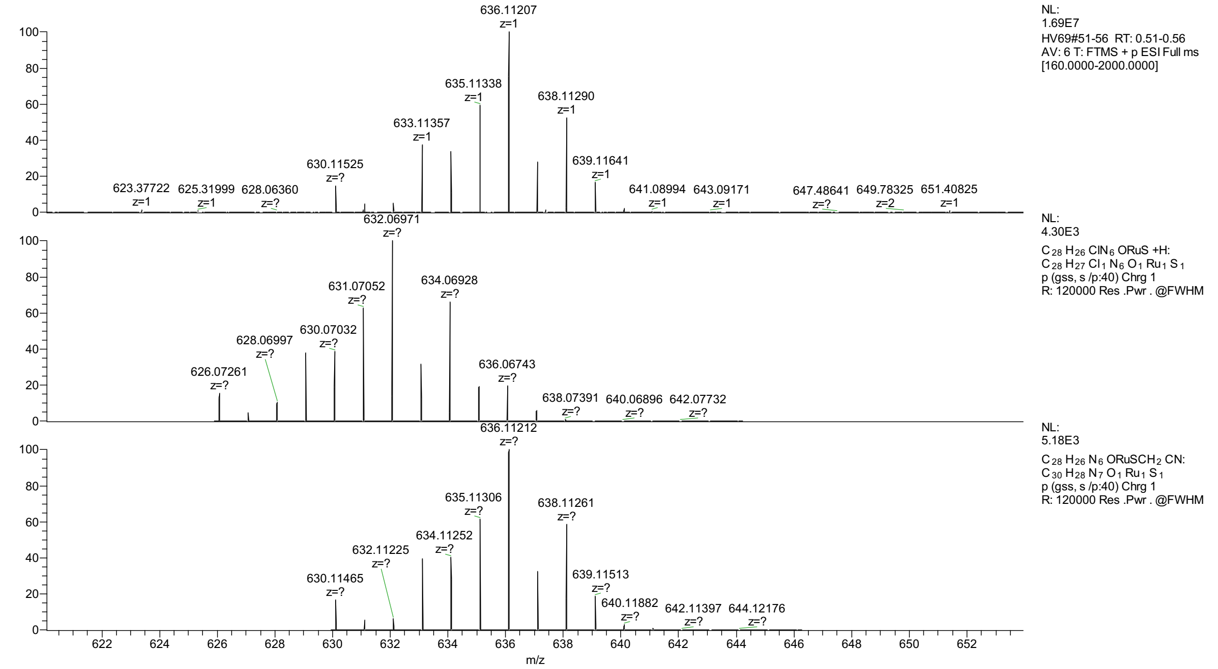

Figure S19. Isotopic distribution of [**3**]Cl. Top measured, middle calculated for $[M+H-Cl]^+$ and bottom calculated for $[M+MeCN–2Cl-H]^+$.

1.3.4 [Ru(biqbpy)(MTSO)(Cl)]Cl [4]Cl

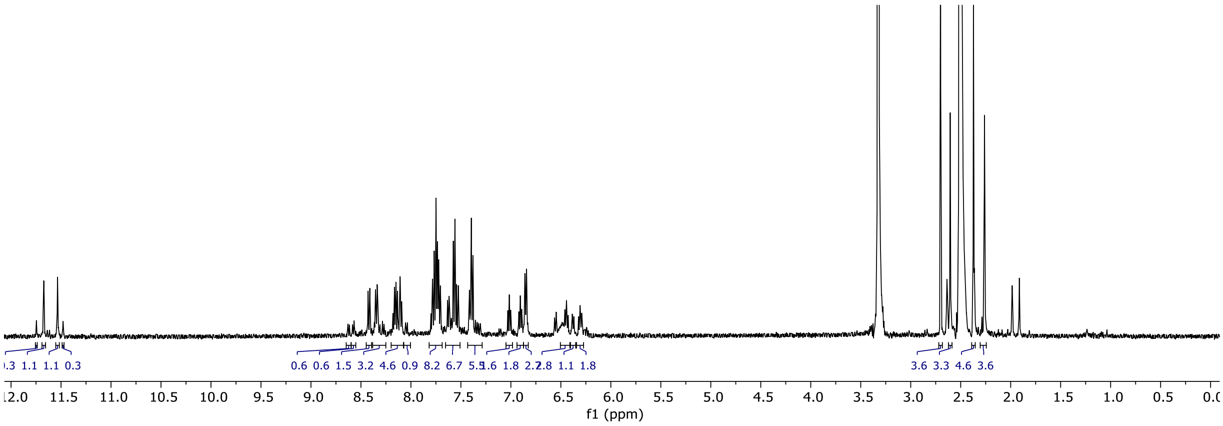

Figure S20. ^{1}H-NMR of [**4**]Cl in $CDCl_3$.

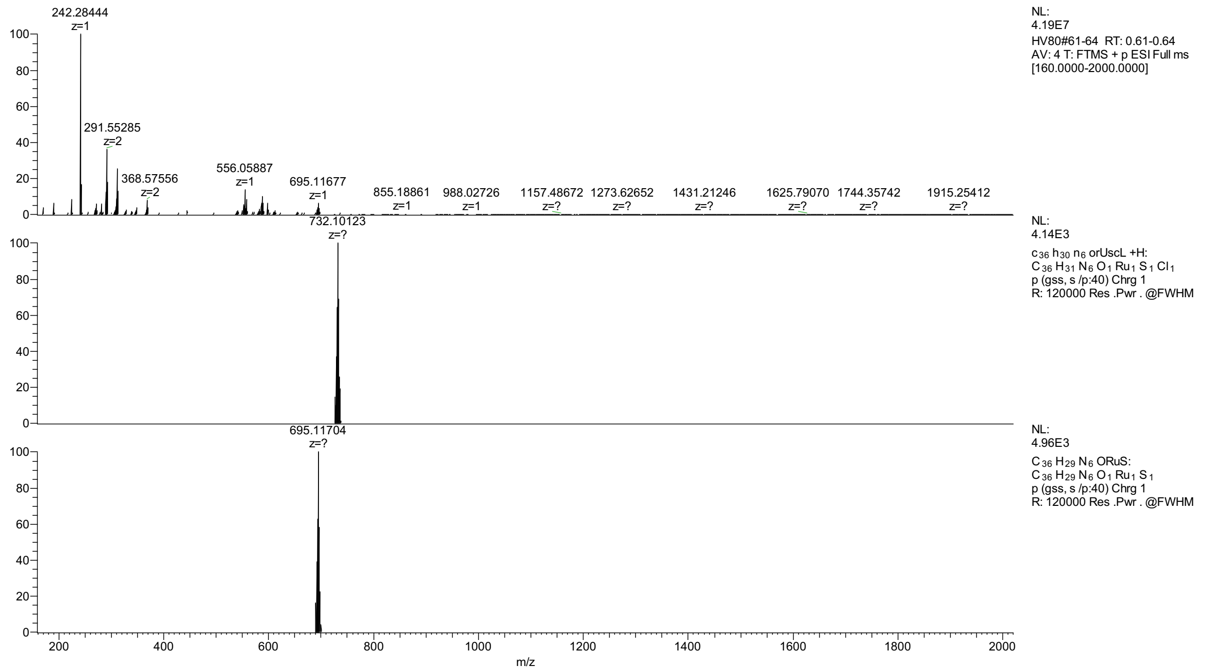

Figure S21. HR-MS of [**4**]Cl. Top measured, middle calculated for $[M+H-Cl]^+$ and bottom calculated for $[M-2Cl-H]^+$.

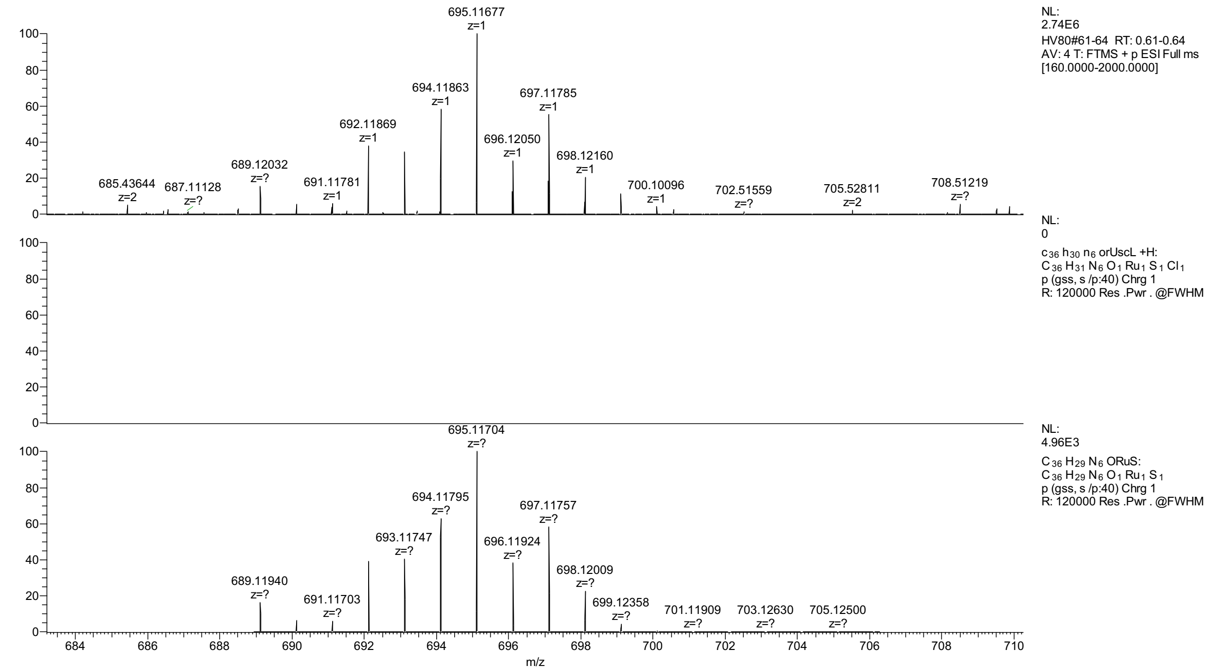

Figure S22. Isotopic distribution of [**2**]Cl. Top measured, bottom calculated for $[M-2Cl-H]^{+}$.

1.3.5 [Ru(biqbpy)(EtOPy)$_2$](PF$_6$)$_2$ [5](PF$_6$)$_2$

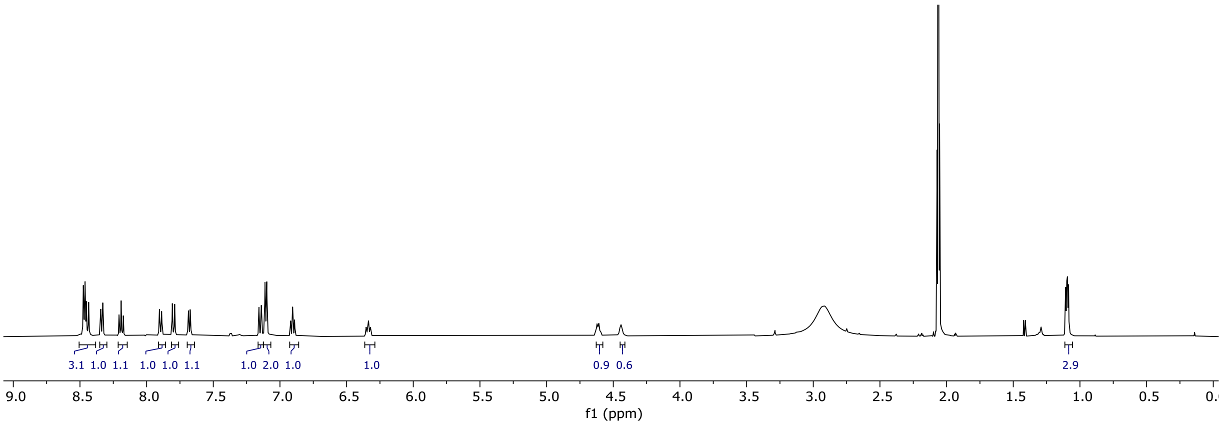

Figure S23. ^{1}H-NMR of [**5**](PF$_6$)$_2$ in aceton-d_6

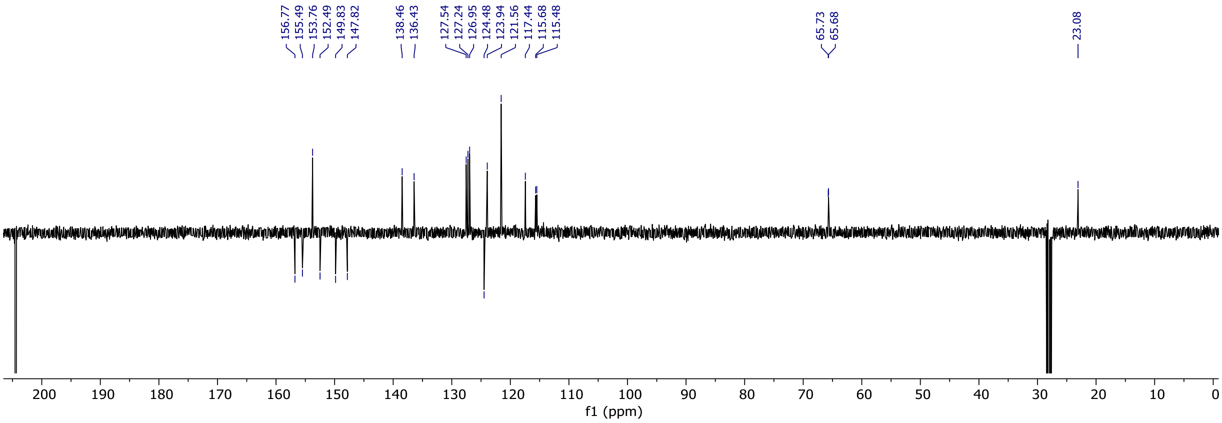

Figure S24. ^{13}C-APT-NMR of [**5**](PF$_6$)$_2$ in aceton-d_6.

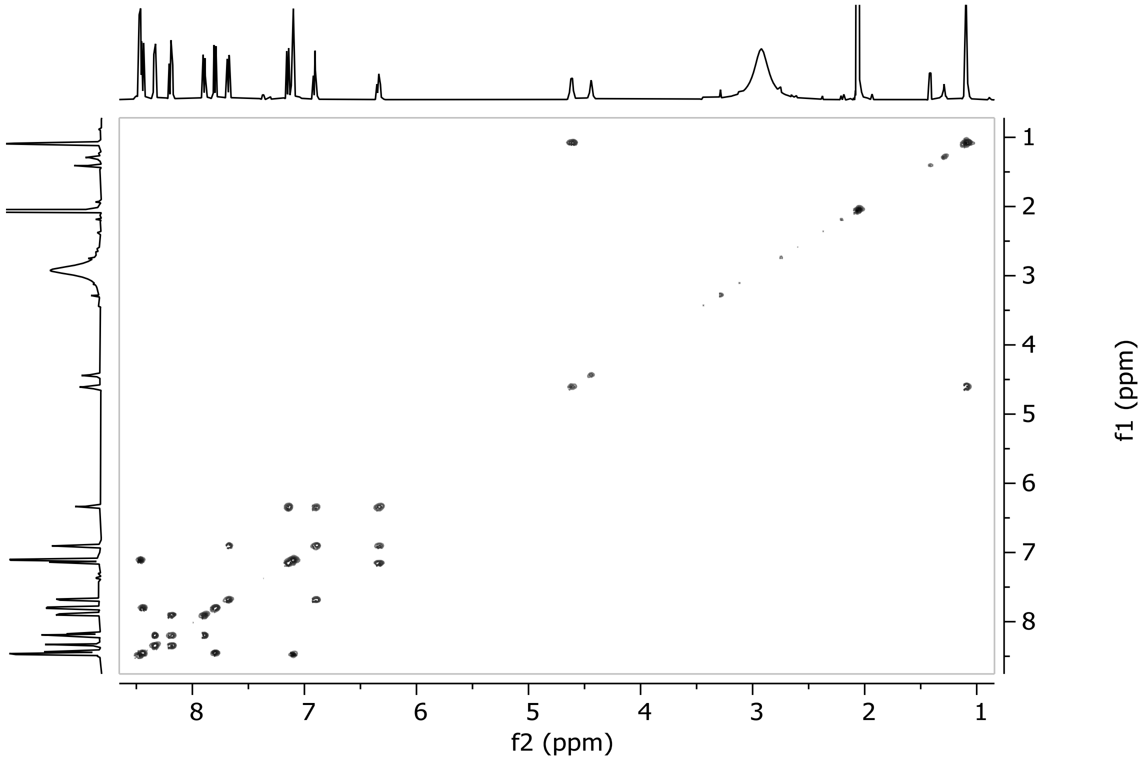

Figure S25. ^{1}H-^{1}H-Cosy-NMR of [**5**]$(PF_6)_2$ in aceton-d_6.

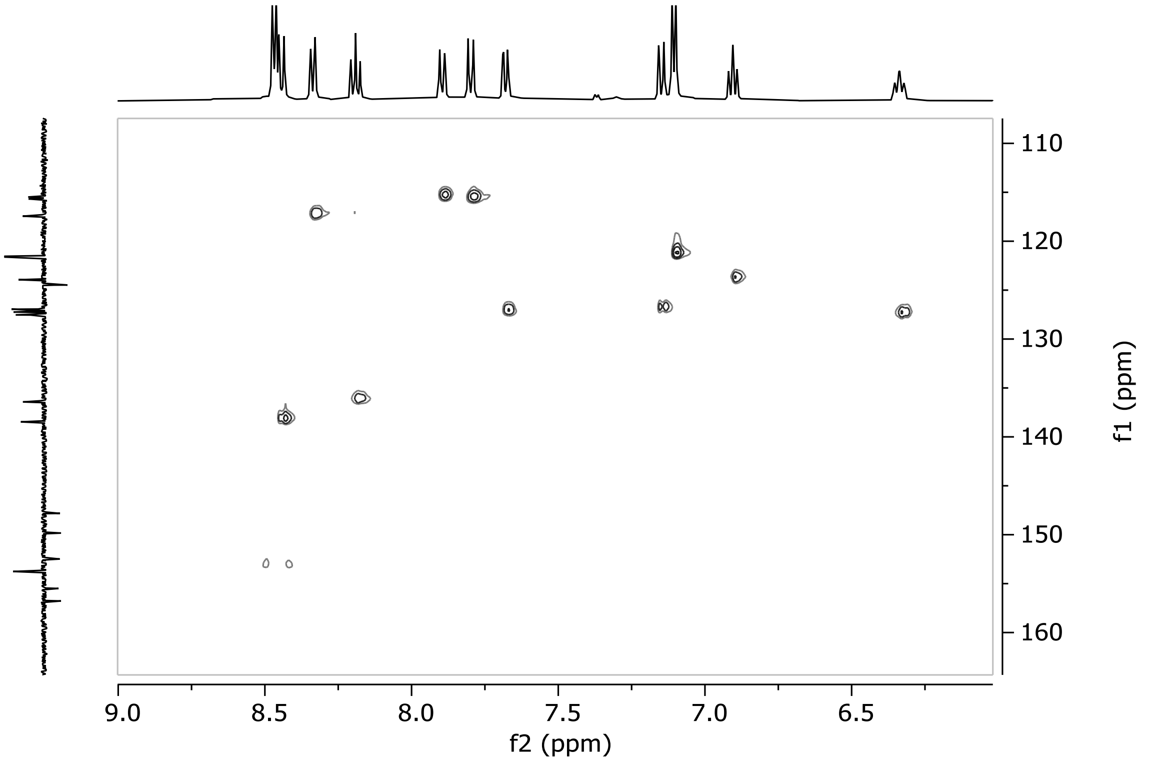

Figure S26. ^{1}H-^{13}C-HSQC of [**5**]$(PF_6)_2$ in aceton-d_6.

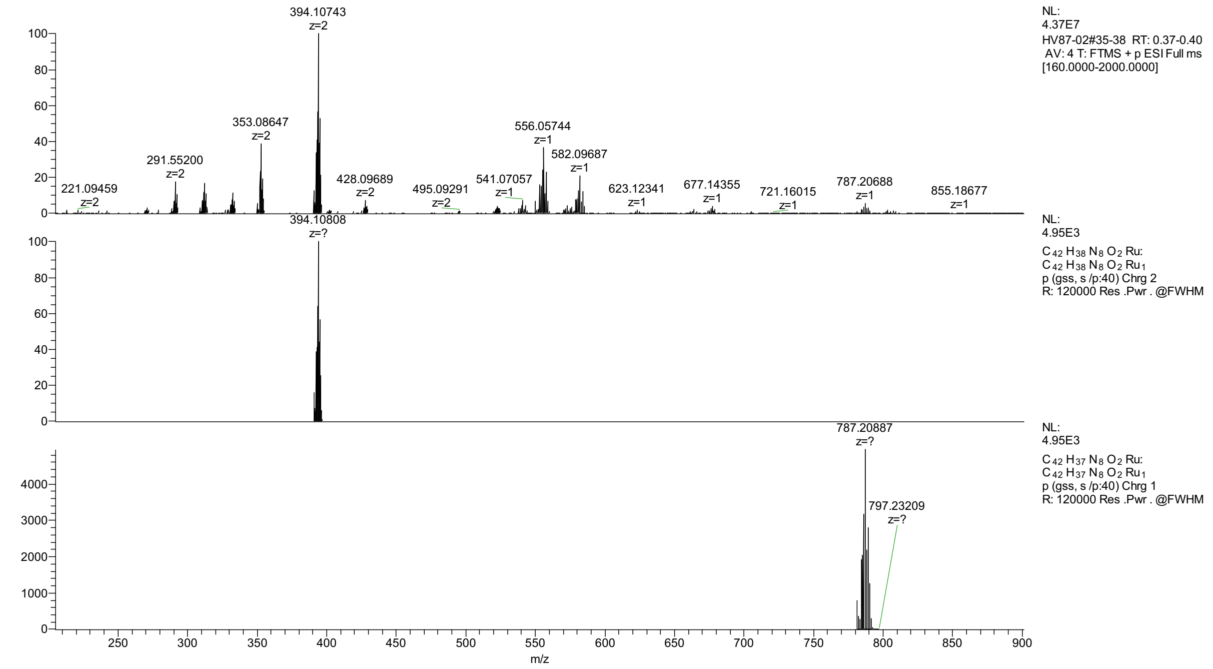

Figure S27. HR-MS of [**5**](PF_6)$_2$. Top measured, middle calculated for [M-2(PF_6)]$^{2+}$ and bottom calculated for [M-H-2(PF_6)]$^+$.

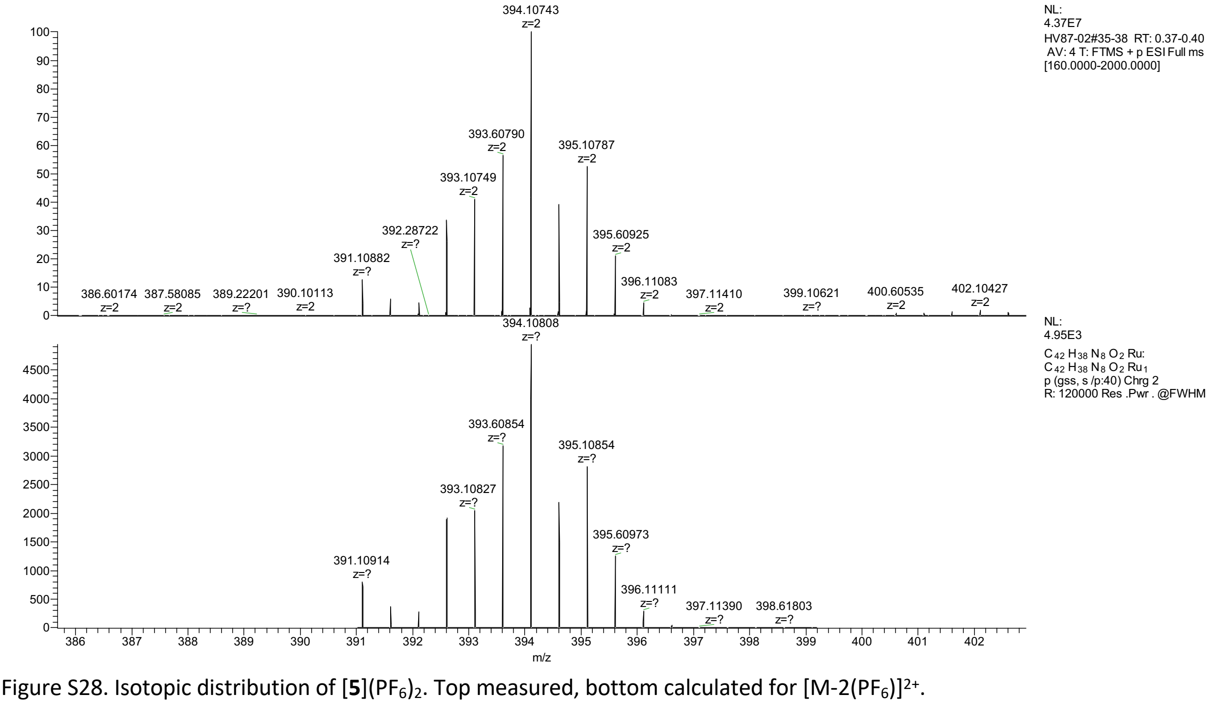

Figure S28. Isotopic distribution of [**5**](PF_6)$_2$. Top measured, bottom calculated for [M-2(PF_6)]$^{2+}$.

1.3.6 [Ru(macro)(DMSO)Cl]Cl [6]Cl

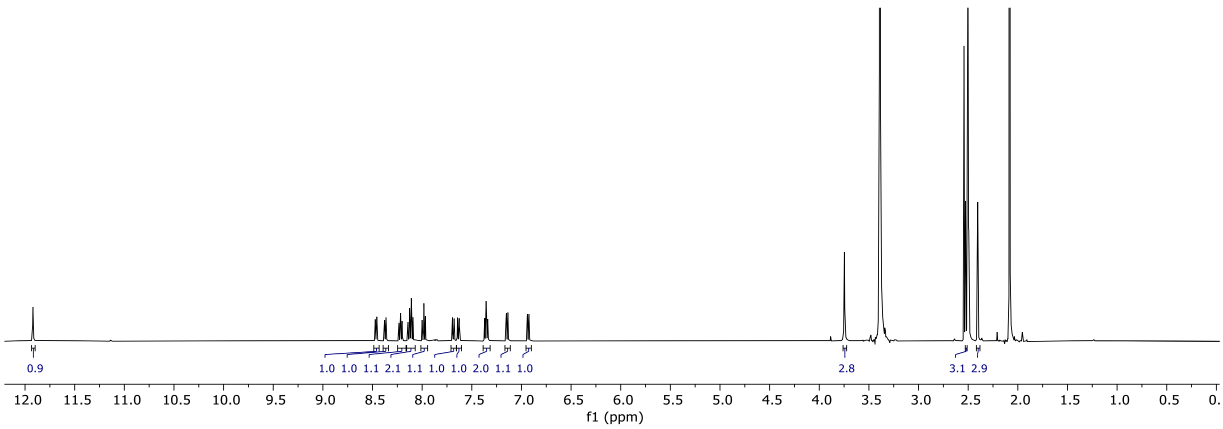

Figure S29. ¹H-NMR of [**6**]Cl in DMSO-d_6.

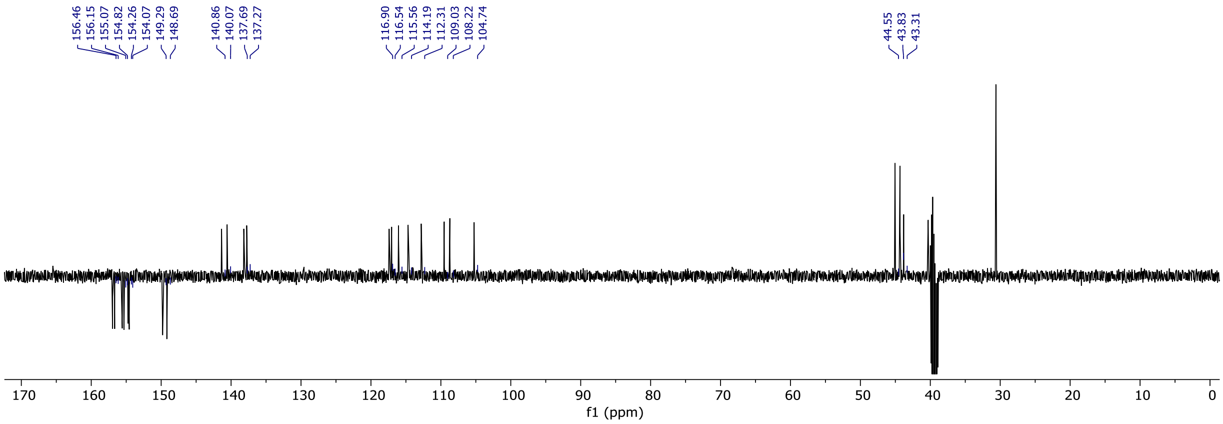

Figure S30. ¹³C-APT-NMR of [**6**]Cl in DMSO-d_6.

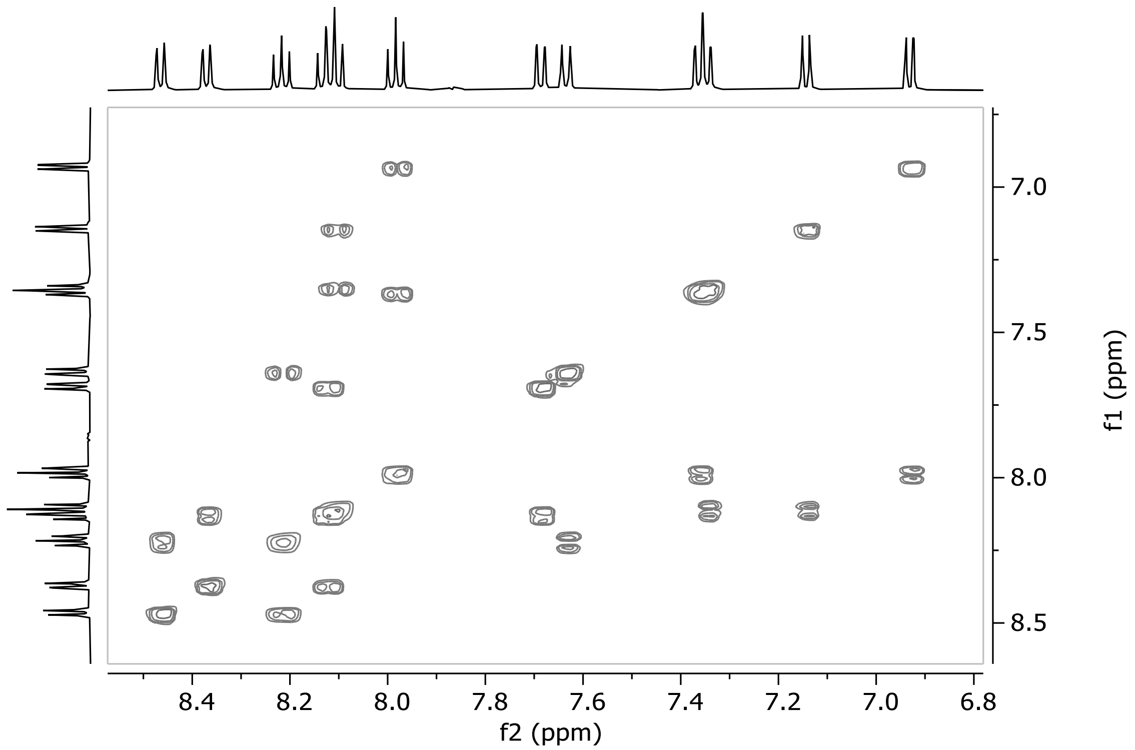

Figure S31. ¹H-¹H-Cosy-NMR of [**6**]Cl in DMSO-d_6.

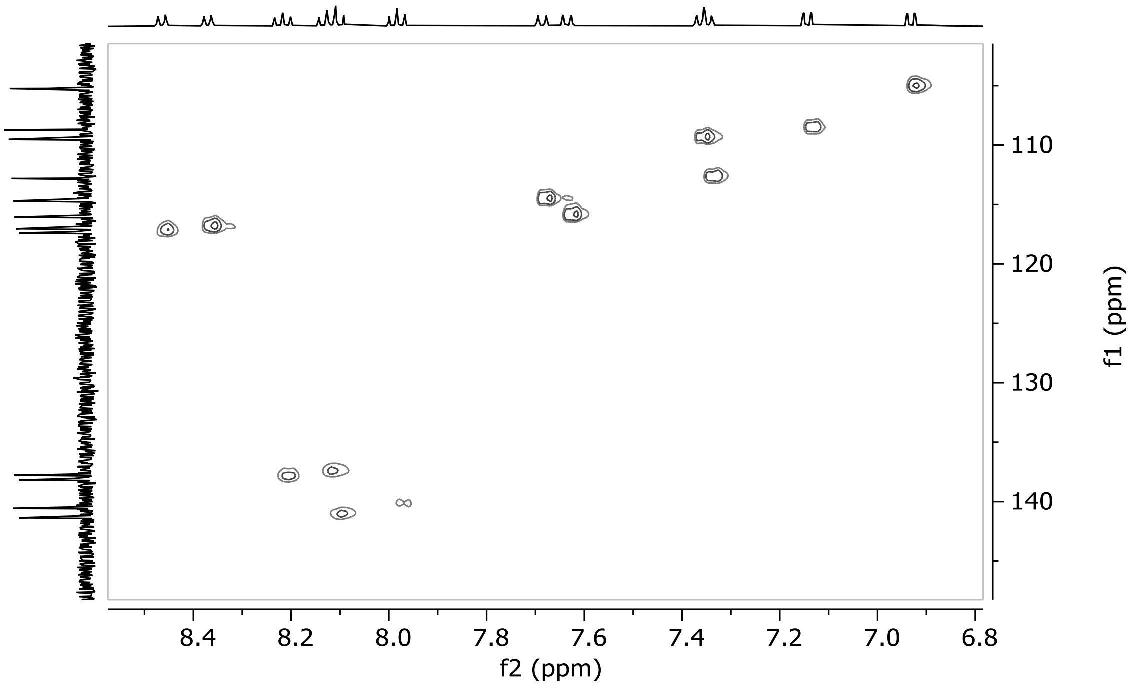

Figure S32. ^{1}H-^{13}C-HSQC of [**6**]Cl in DMSO-d_6.

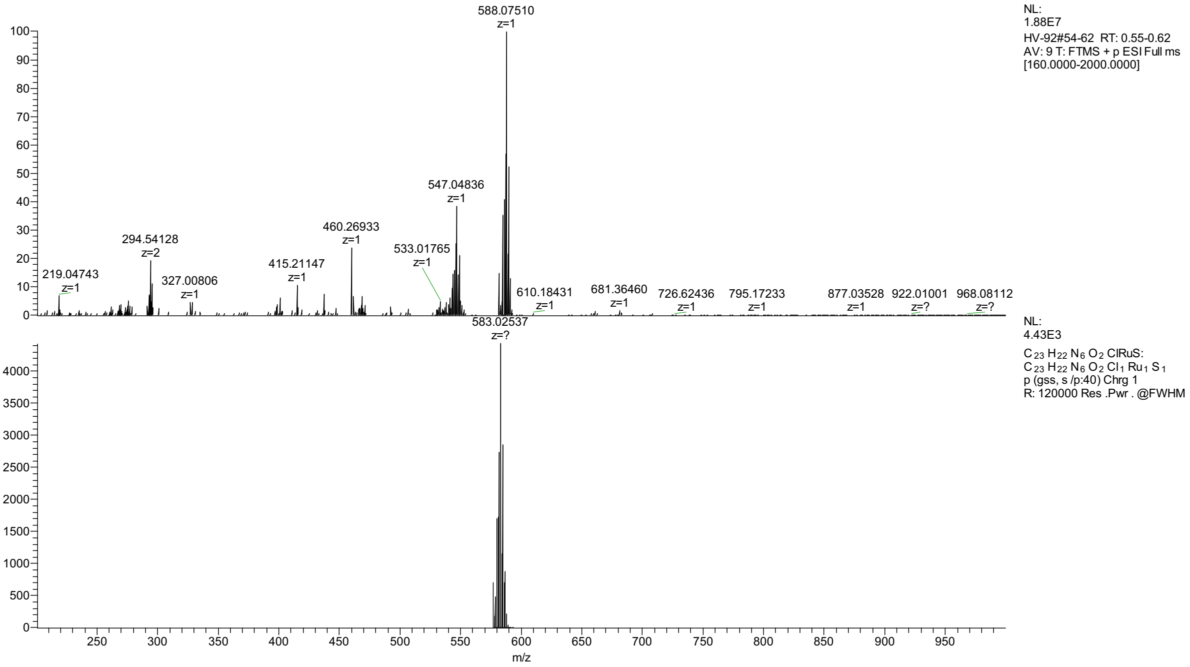

Figure S33. HR-MS of [**6**]Cl. Top measured, bottom calculated for [M–Cl]$^{+}$.

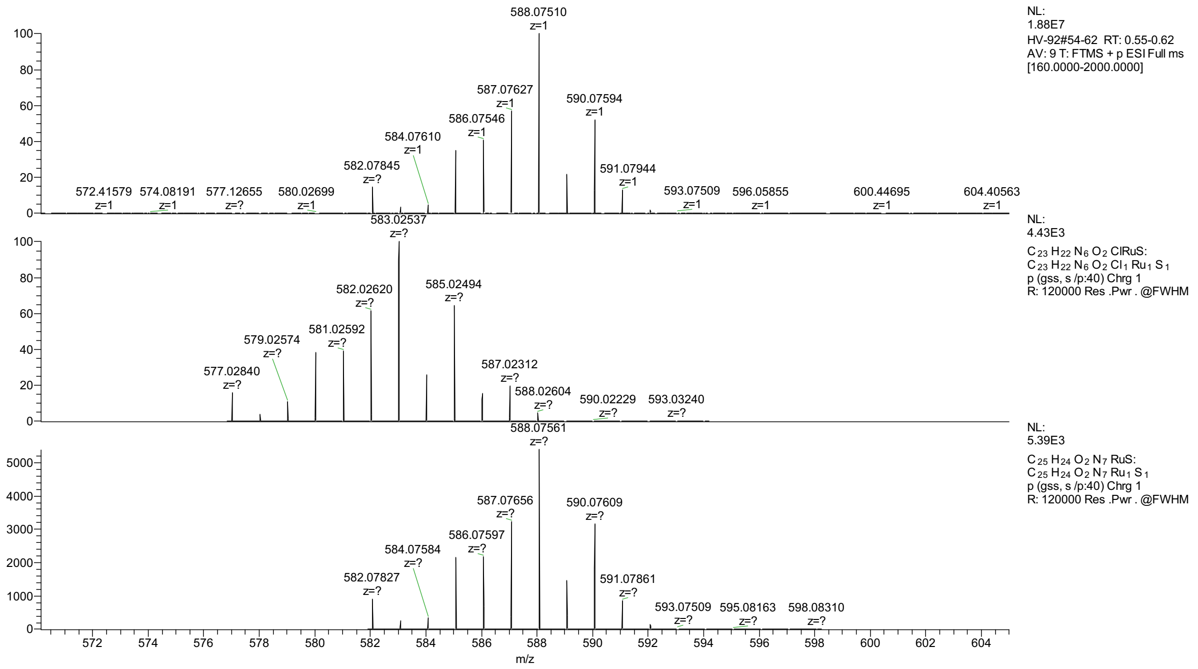

Figure S34. Isotopic distribution of [**6**]Cl. Top measured, middle calculated for $[M{-}Cl]^+$ and bottom calculated for $[M{+}MeCN{-}2Cl{-}H]^+$.

1.3.7 [Ru(macro)(MTSO)Cl]Cl [7]Cl

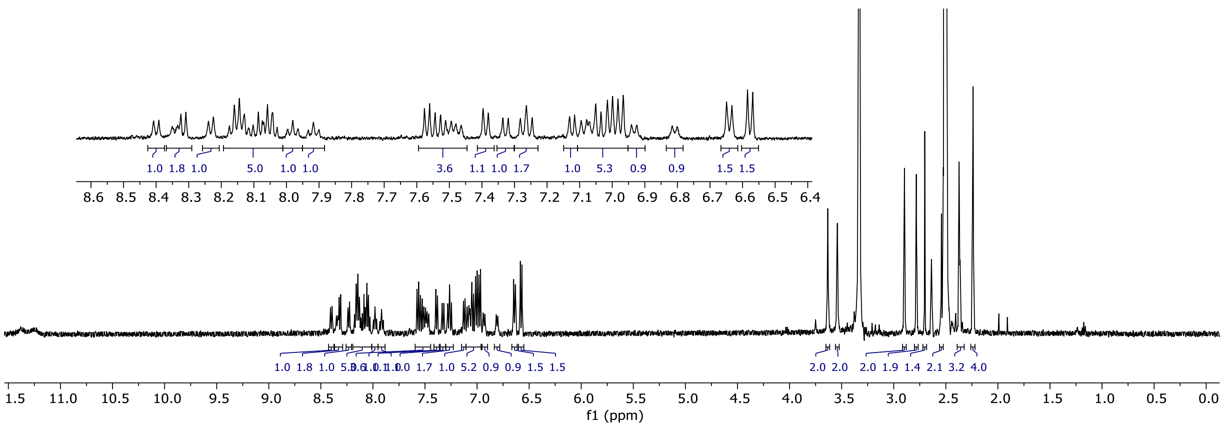

Figure S35. ^{1}H-NMR of [**7**]Cl in DMSO-d_6.

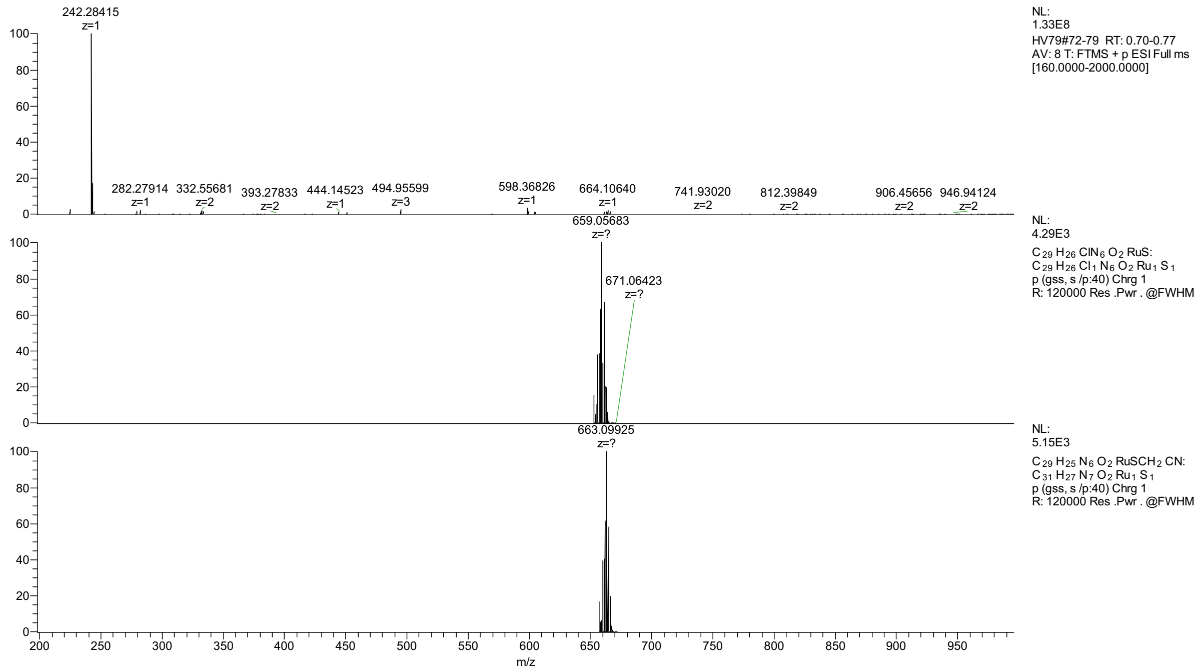

Figure S36. HR-MS of [**7**]Cl. Top measured, middle calculated for $[M-Cl]^+$ and bottom calculated for $[M+MeCN-2Cl-H]^+$.

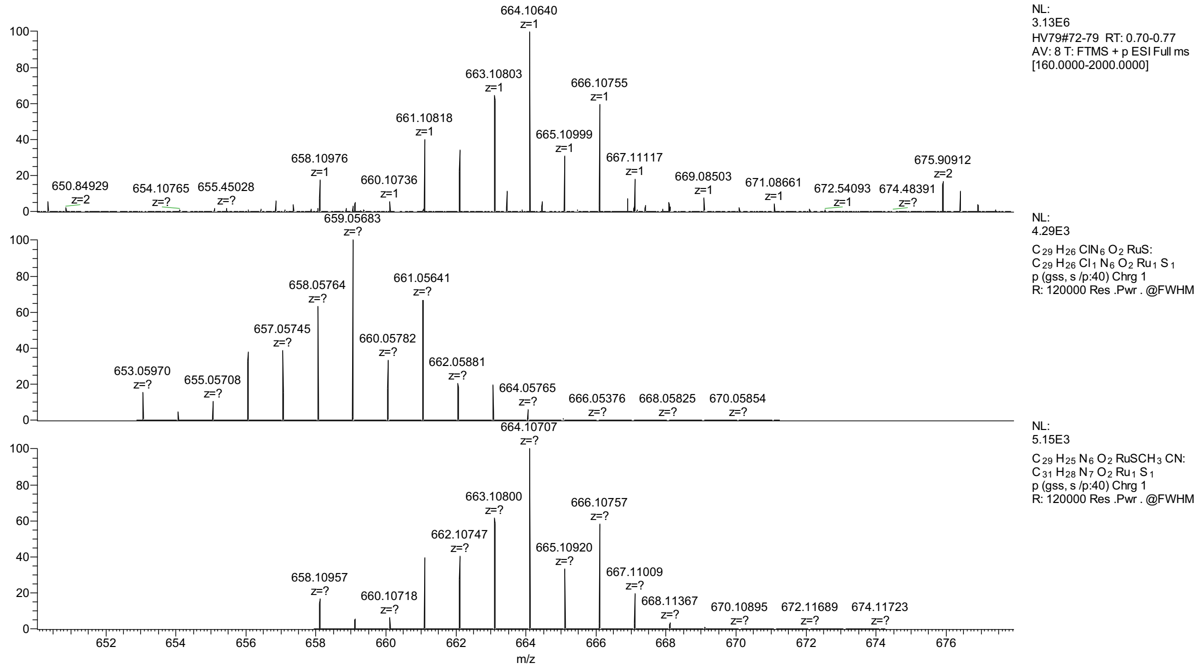

Figure S37. Isotopic distribution of [**7**]Cl. Top measured, middle calculated for $[M-Cl]^+$ and bottom calculated for $[M+MeCN-2Cl-H]^+$.

1.3.8 [Pd(OMe-babppy)]$(Cl)_2$ [8]Cl

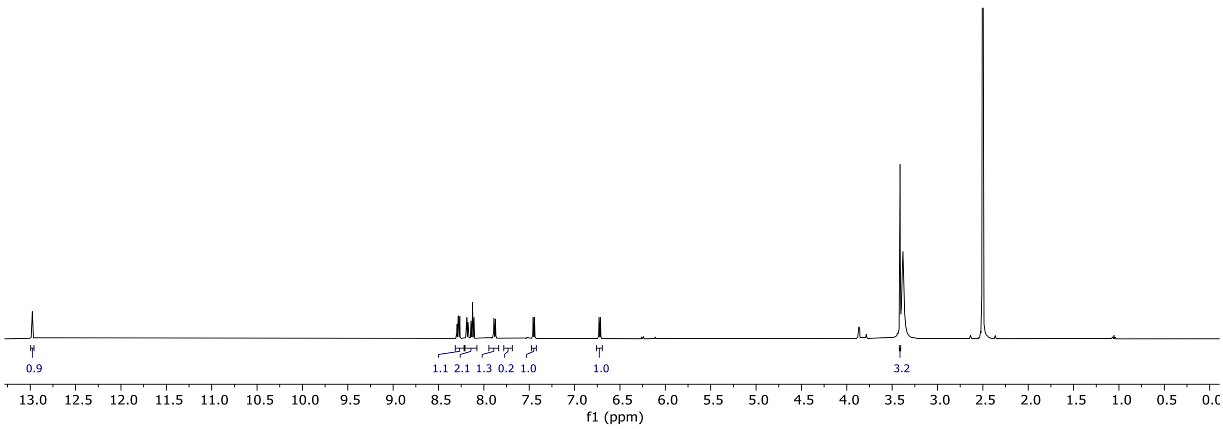

Figure S38. ^{1}H-NMR of [**8**]Cl in DMSO-d_6.

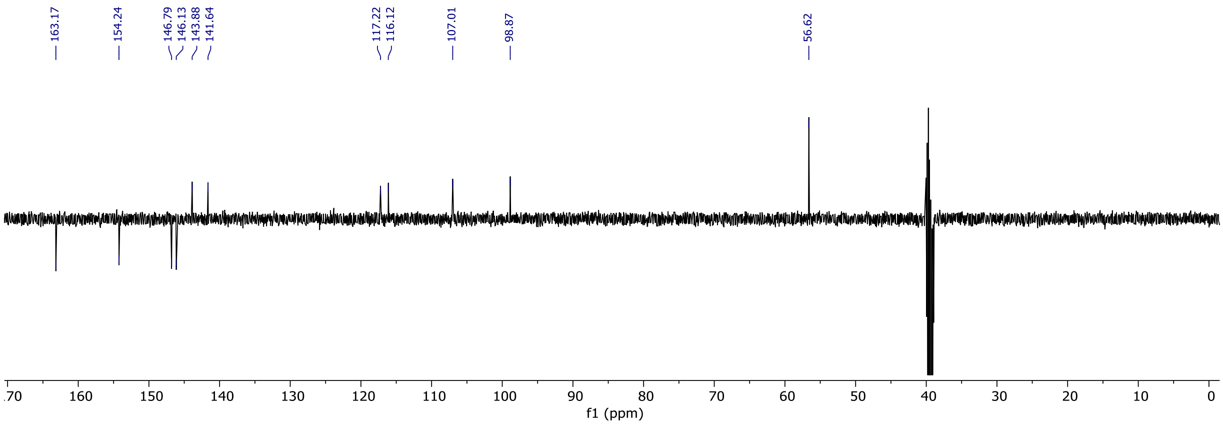

Figure S39. ^{13}C-APT-NMR of [**8**]Cl in DMSO-d_6.

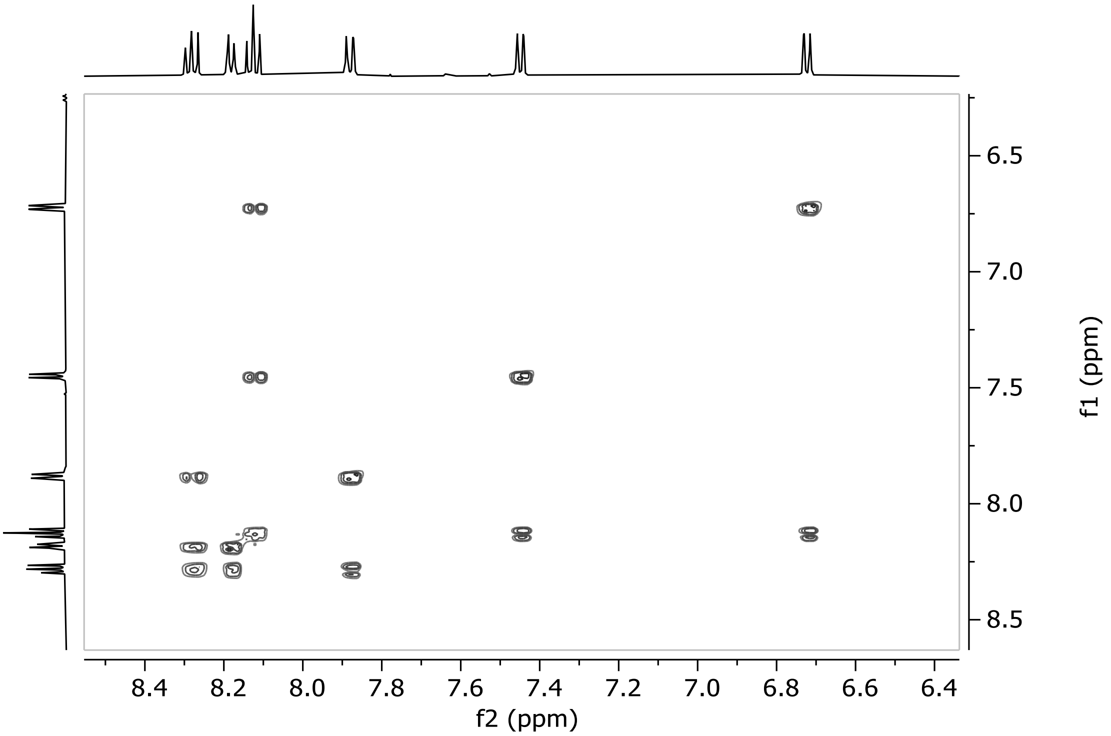

Figure S40. ^{1}H-^{1}H-Cosy-NMR of [**8**]Cl in DMSO-d_6.

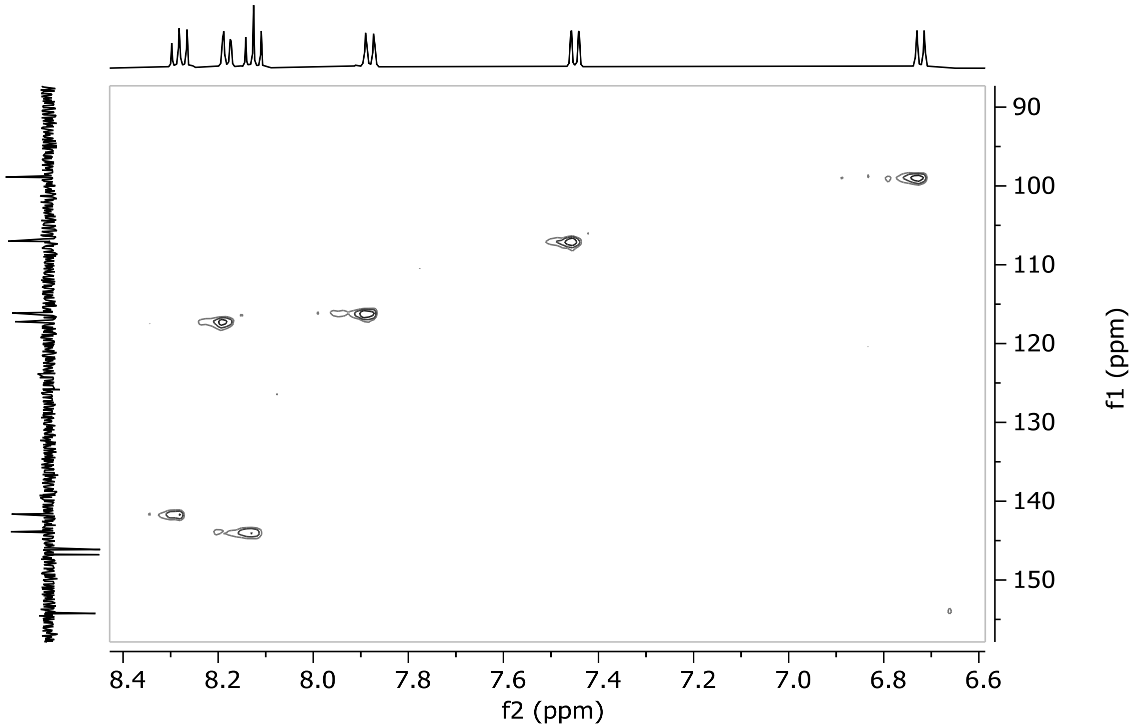

Figure S41. ^{1}H-^{13}C-HSQC of [**8**]Cl in DMSO-d_6.

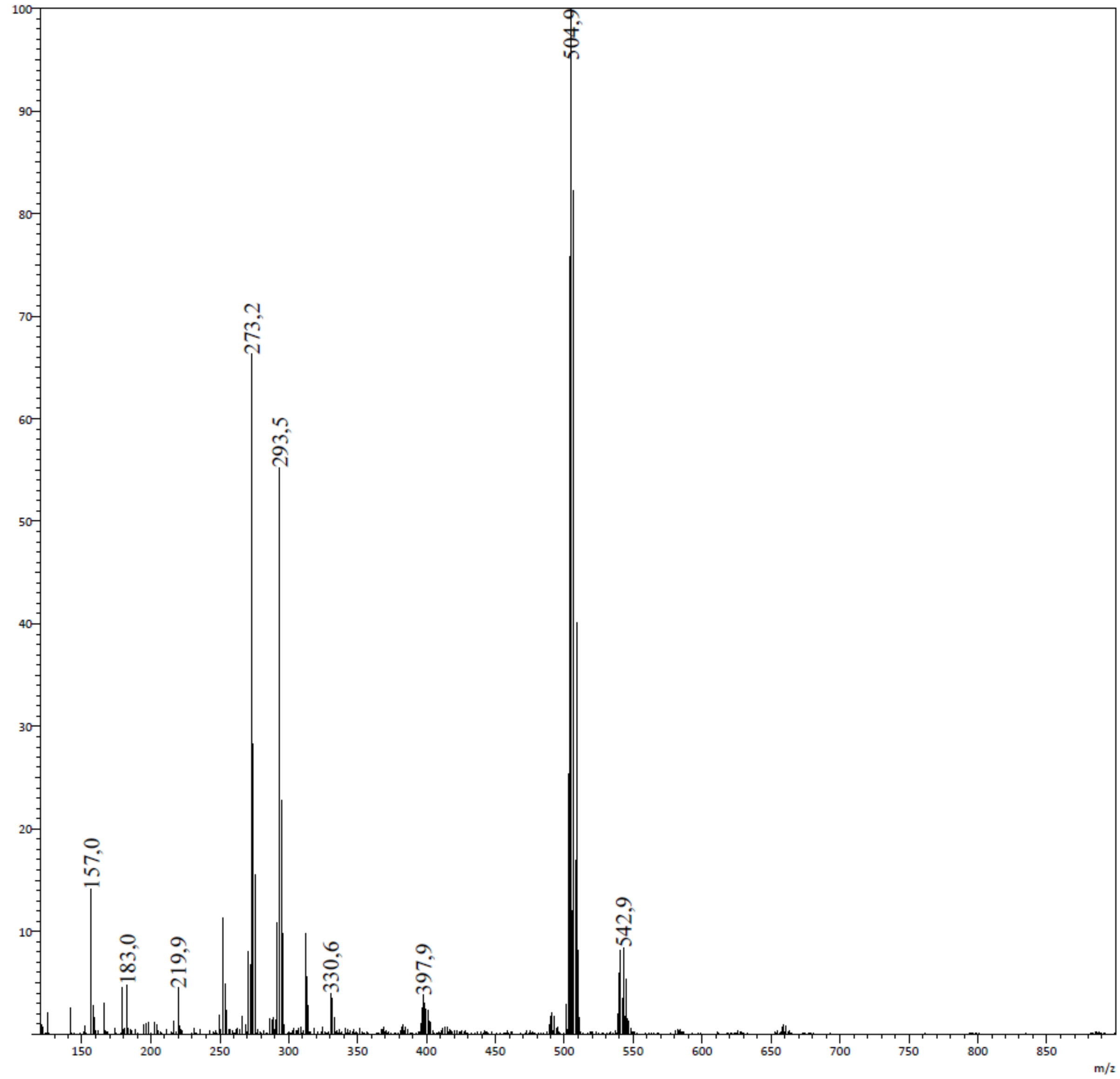

Figure S42. ES MS of [8]Cl.

1.3.9 [Rh(OMe-bapbpy)Cl$_2$](Cl)

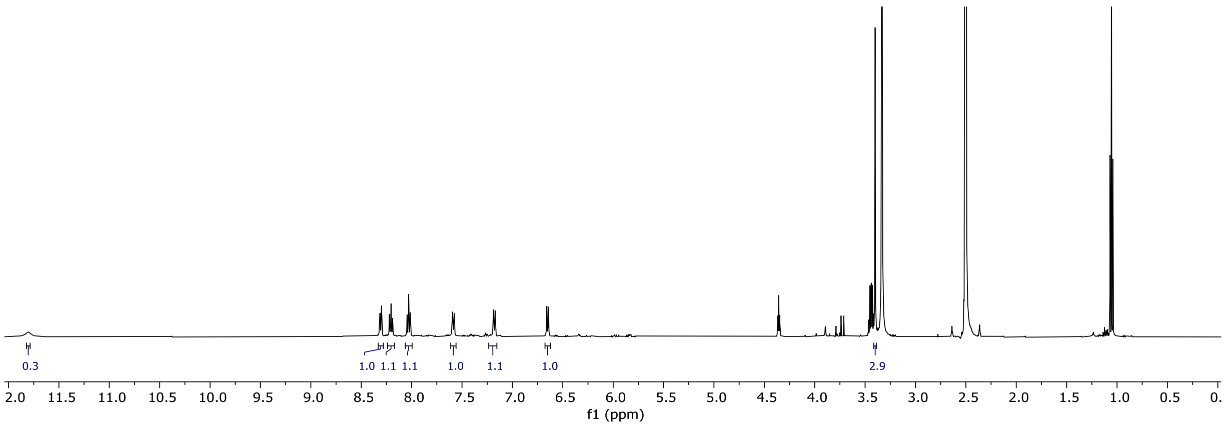

Figure S43. ^{1}H-NMR of [Rh(OMe-bapbpy)(Cl)$_2$](Cl) **i**nDMSO-d_6.

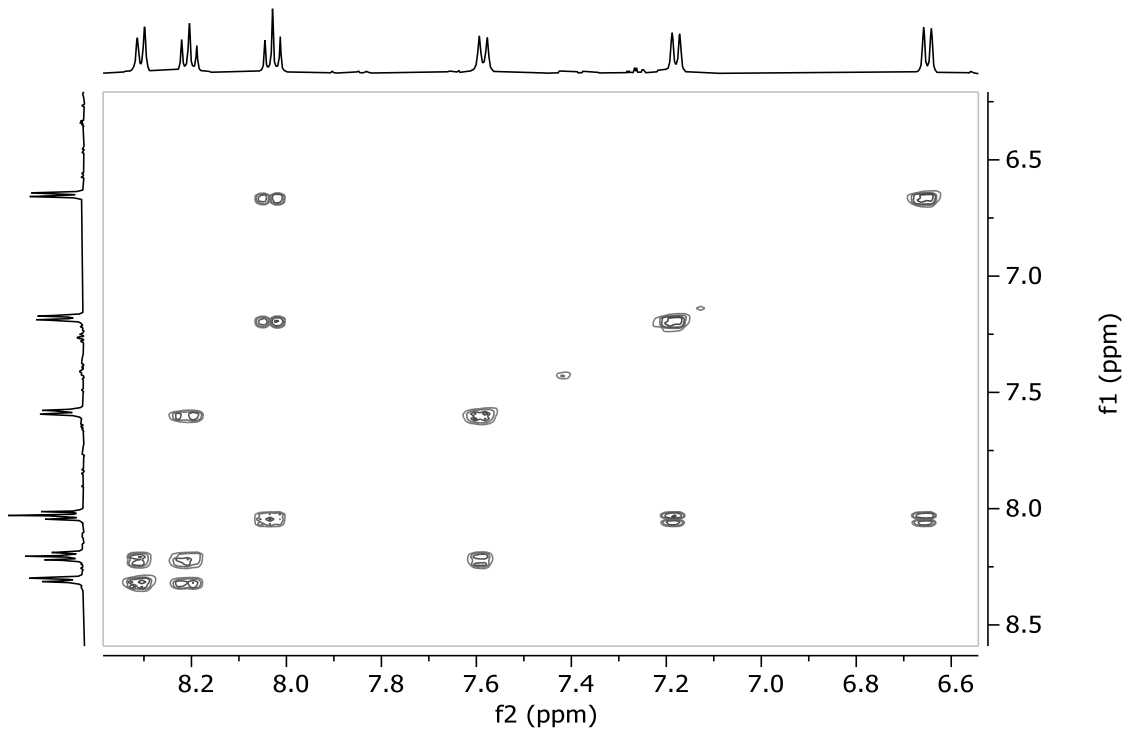

Figure S44. ^{1}H-^{1}H-Cosy-NMR of $[Rh(OMe\text{-}bapbpy)(Cl)_2](Cl)$ in DMSO-d_6.

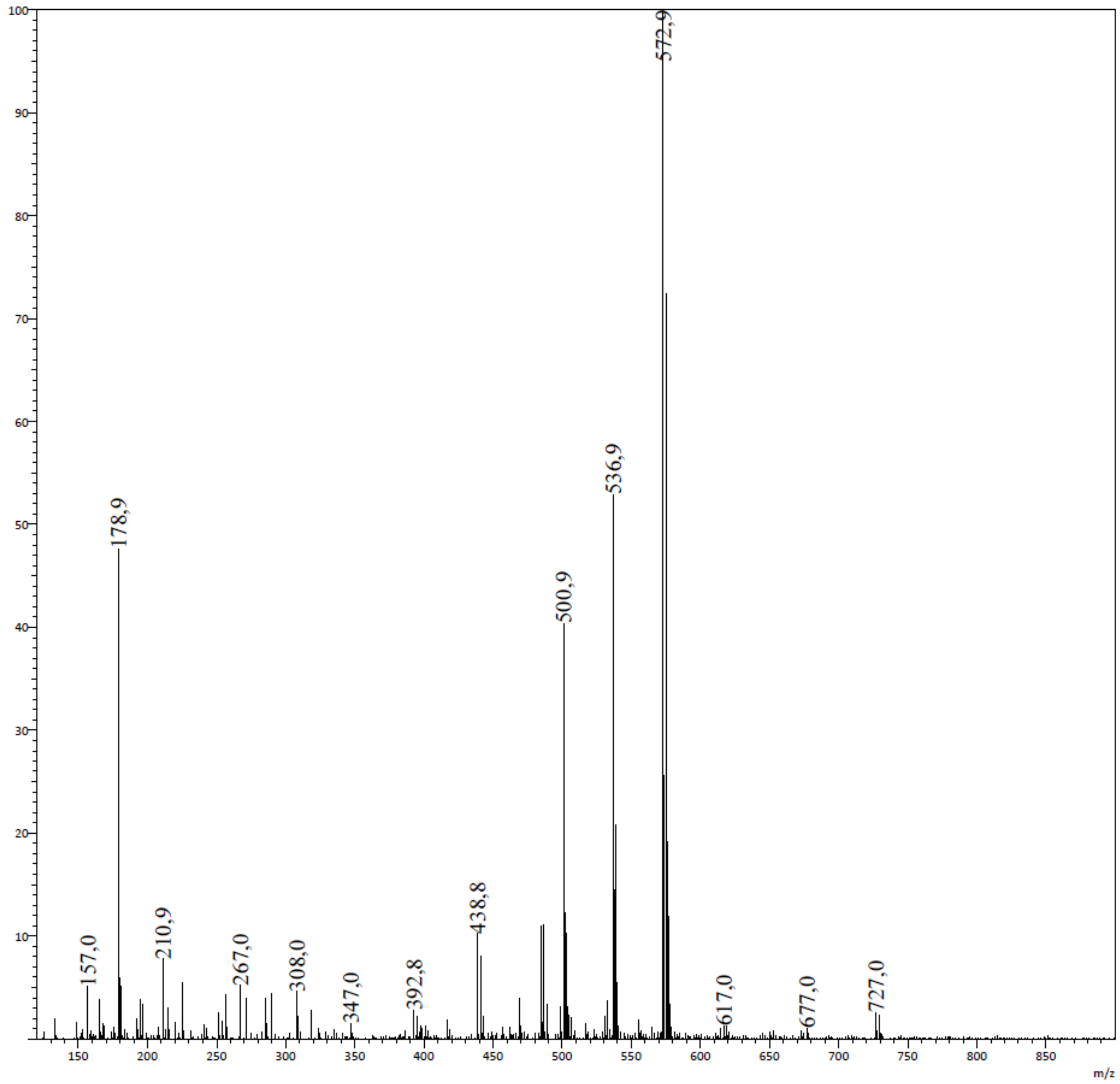

Figure S45. ES MS of [Rh(OMe-bapbpy)(Cl)$_2$]Cl·

1.4 Crystal structures

1.4.1 [Ru(bapbpy)(MTSO)(Cl)]PF_6, [3]PF_6

All reflection intensities were measured at 110(2) K using a SuperNova diffractometer (equipped with Atlas detector) with Mo *K*α radiation (λ = 0.71073 Å) under the program CrysAlisPro (Version CrysAlisPro 1.171.39.29c, Rigaku OD, 2017). The same program was used to refine the cell dimensions and for data reduction. The structure was solved with the program SHELXS-2018/3 (Sheldrick, 2018) and was refined on F^2 with SHELXL-2018/3.[1] Numerical absorption correction based on gaussian integration over a multifaceted crystal model was applied using CrysAlisPro. The temperature of the data collection was controlled using the system Cryojet (manufactured by Oxford Instruments). The H atoms were placed at calculated positions (unless otherwise specified) using the instructions AFIX 43 or AFIX 137 with isotropic displacement parameters having values 1.2 or 1.5 U_{eq} of the attached C atoms. The H atoms attached to N2X and N5X (X = A, B) were found from difference Fourier maps, and their coordinates were restrained pseudofreely using the DFIX instruction in order to keep the N–H distances within an acceptable range. The structure is partly disordered.

The two PF_6^- counterions are found disordered over two orientations, and the occupancy factors of the major components of the disorder 0.872(7) and 0.728(15). The asymmetric unit also contains some amount of very disordered lattice solvent molecules (most likely MeOH), and their contribution has been removed using the SQUEEZE procedure in Platon.[2]

The absolute configuration has been established by anomalous-dispersion effects in diffraction measurements on the crystal, and the Flack parameter refines to 0.01(4).

ADDSYM suggests a missed inversion center. However, a refinement in the *P*-1 space group constrains the Ru complex and counterions to be more disordered. The fact that the value of the Flack parameter refines close to zero suggests that the *P*1 refinement is indeed correct.

Table S1. Crystallographic Data for [3]PF_6

	**[3]PF_6**
Crystal data	
Chemical formula	$C_{28}H_{26}ClN_6ORuS{\cdot}F_6P$
M_r	776.10
Crystal system, space group	Triclinic, *P*1
Temperature (K)	110
a, *b*, *c* (Å)	11.6908 (4), 12.7070 (4), 13.4982 (4)
α, β, γ (°)	112.889 (3), 115.601 (3), 92.947 (2)
V (Å^3)	1606.79 (10)
Z	2
Radiation type	Mo *K*α
μ (mm^{-1})	0.75
Crystal size (mm)	0.20 × 0.11 × 0.04
Data collection	
Diffractometer	SuperNova, Dual, Cu at zero, Atlas
Absorption correction	Gaussian *CrysAlis PRO* 1.171.39.29c (Rigaku Oxford Diffraction, 2017) Numerical absorption correction based on gaussian integration over a multifaceted crystal model Empirical absorption correction using spherical harmonics, implemented in SCALE3 ABSPACK scaling algorithm.
T_{min}, T_{max}	0.764, 1.000
No. of measured, independent and observed [$I > 2\sigma(I)$] reflections	25098, 13595, 12081
R_{int}	0.028
$(\sin \theta/\lambda)_{max}$ (Å^{-1})	0.650
Refinement	
$R[F^2 > 2\sigma(F^2)]$, $wR(F^2)$, S	0.042, 0.097, 1.04
No. of reflections	13595
No. of parameters	926
No. of restraints	481
H-atom treatment	H atoms treated by a mixture of independent and constrained refinement
$\Delta\rho_{max}$, $\Delta\rho_{min}$ (e Å^{-3})	1.11, -0.51
Absolute structure	Refined as an inversion twin.
Absolute structure parameter	0.01 (4)

1.4.2 [Ru(biqbpy)(EtOHPy)$_2$](PF$_6$)$_2$, [5](PF$_6$)$_2$

All reflection intensities were measured at 173(2) K using a SuperNova diffractometer (equipped with Atlas detector) with Cu Kα radiation (λ = 1.54178 Å) under the program CrysAlisPro (Version CrysAlisPro 1.171.39.29c, Rigaku OD, 2017). The same program was used to refine the cell dimensions and for data reduction. The structure was solved with the program SHELXS-2018/3 (Sheldrick, 2018) and was refined on $F^2$with SHELXL-2018/3.[1] Analytical numeric absorption correction using a multifaceted crystal model was performed using CrysAlisPro. The temperature of the data collection was controlled using the system Cryojet (manufactured by Oxford Instruments). The H atoms were placed at calculated positions using the instructions AFIX 13, AFIX 43 or AFIX 137 with isotropic displacement parameters having values 1.2 or 1.5 Ueq of the attached C or N atoms. The H atoms attached to O1X and O2X (X = A–D), O1G, O2E, O2H and O1S could not be retrieved reliably from difference Fourier maps.

The structure is significantly disordered. The asymmetric contains overall four crystallographically independent Ru cations, eight PF6–counterions (see below for further details), and some amount of lattice solvent molecules. Three 1-hydroxyethyl groups are disordered over two orientations. Overall, there are eight PF6–counterions: P2, P3, P4 and P5 are ordered and fully occupied, P7/P7' and P8/P8'are disordered over two orientations but their sites are fully occupied, and P1, P6, P9, P10 are disordered and are half occupied as they are found at sites of twofold axial symmetry. All occupancy factors of the major / minor components of the disorder can be retrieved from the final .cif files. The asymmetric unit also contains some amount of lattice solvent molecules. One MeOH lattice solvent molecule was modeled as ordered but the remaining lattice solvent molecules (most likely Et2O) are found very disordered, and their contribution was removed from the final refinement using the SQUEEZE procedure in Platon.[2]

The absolute configuration has been established by anomalous-dispersion effects in diffraction measurements on the crystal, and the Flack and Hooft parameters refine to 0.094(11) and 0.071(6).

Table S2. Crystallographic Data for [5](PF_6)$_2$

	**[5](PF_6)$_2$**
Crystal data	
Chemical formula	4($C_{42}H_{36}N_8O_2Ru$)·8(F_6P)·CH_3O
M_r	4334.22
Crystal system, space group	Orthorhombic, $P2_12_12$
Temperature (K)	173
a, b, c (Å)	27.5618 (4), 43.8925 (6), 17.21821 (17)
V (Å^3)	20829.8 (5)
Z	4
Radiation type	Cu $K\alpha$
μ (mm^{-1})	3.77
Crystal size (mm)	0.41 × 0.04 × 0.04
Data collection	
Diffractometer	SuperNova, Dual, Cu at zero, Atlas
Absorption correction	Analytical<br>*CrysAlis PRO* 1.171.42.49 (Rigaku Oxford Diffraction, 2022) Analytical numeric absorption correction using a multifaceted crystal model based on expressions derived by R.C. Clark & J.S. Reid. (Clark, R. C. & Reid, J. S. (1995). Acta Cryst. A51, 887-897) Empirical absorption correction using spherical harmonics, implemented in SCALE3 ABSPACK scaling algorithm.
T_{min}, T_{max}	0.456, 0.894
No. of measured, independent and observed [$I > 2\sigma(I)$] reflections	112040, 37331, 29337
R_{int}	0.066
$(\sin \theta/\lambda)_{max}$ (Å^{-1})	0.598
Refinement	
$R[F^2 > 2\sigma(F^2)]$, $wR(F^2)$, S	0.060, 0.147, 1.03
No. of reflections	37331
No. of parameters	2782
No. of restraints	2198
H-atom treatment	H-atom parameters constrained
	$w = 1/[\sigma^2(F_o^2) + (0.0526P)^2 + 28.3501P]$<br>where $P = (F_o^2 + 2F_c^2)/3$
$\Delta\rho_{max}$, $\Delta\rho_{min}$ (e Å^{-3})	1.47, -0.77
Absolute structure	Refined as an inversion twin.
Absolute structure parameter	0.094 (11)

1.4.3 [Ru(macro)(DMSO)(Cl)]OTf, [6]OTf

All reflection intensities were measured at 110(2) K using a SuperNova diffractometer (equipped with Atlas detector) with Cu *K*α radiation (λ = 1.54178 Å) under the program CrysAlisPro (Version CrysAlisPro 1.171.39.29c, Rigaku OD, 2017). The same program was used to refine the cell dimensions and for data reduction. The structure was solved with the program SHELXS-2018/3 (Sheldrick, 2018) and was refined on F^2 with SHELXL-2018/3.[1] Analytical numeric absorption correction using a multifaceted crystal model was applied using CrysAlisPro. The temperature of the data collection was controlled using the system Cryojet (manufactured by Oxford Instruments). The H atoms were placed at calculated positions using the instructions AFIX 43 or AFIX 137 with isotropic displacement parameters having values 1.2 or 1.5 U_{eq} of the attached C or N atoms. The structure is partly disordered.

The two triflate counterions are found to be disordered over either two of three orientations. All occupancy factors can be retrieved from the final .cif file.

The crystal lattice contains some remaining amount of very disordered lattice solvent molecules, and their contribution has been removed using the SQUEEZE procedure in Platon.[2]

The crystal is non-merohedrally twinned. The twin relationship corresponds to a twofold axis along the -0.0001**a*** + 0.8947**b*** + 0.4466**c*** reciprocal direction. The BASF scale factor refines to 0.4065(11).

Table S3. Crystallographic Data for [6]OTf

	**[6]OTf**
Crystal data	
Chemical formula	$C_{23}H_{22}ClN_6O_2RuS \cdot CF_3O_3S$
M_r	732.11
Crystal system, space group	Triclinic, *P*-1
Temperature (K)	110
a, *b*, *c* (Å)	7.8338 (3), 20.5997 (9), 21.2020 (11)
α, β, γ (°)	64.854 (5), 89.610 (3), 87.433 (3)
V (Å^3)	3093.8 (3)
Z	4
Radiation type	Cu *K*α
μ (mm^{-1})	6.71
Crystal size (mm)	0.13 × 0.04 × 0.02
Data collection	
Diffractometer	SuperNova, Dual, Cu at zero, Atlas
Absorption correction	Analytical<br>*CrysAlis PRO* 1.171.40.53 (Rigaku Oxford Diffraction, 2019) Analytical numeric absorption correction using a multifaceted crystal model based on expressions derived by R.C. Clark & J.S. Reid. (Clark, R. C. & Reid, J. S. (1995). Acta Cryst. A51, 887-897) Empirical absorption correction using spherical harmonics, implemented in SCALE3 ABSPACK scaling algorithm.
T_{min}, T_{max}	0.538, 0.876
No. of measured, independent and observed [$I > 2\sigma(I)$] reflections	32634, 12256, 7032
R_{int}	0.078
$(\sin \theta/\lambda)_{max}$ (Å^{-1})	0.598
Refinement	
$R[F^2 > 2\sigma(F^2)]$, $wR(F^2)$, S	0.053, 0.121, 0.77
No. of reflections	12256
No. of parameters	969
No. of restraints	887
H-atom treatment	H-atom parameters constrained
$\Delta\rho_{max}$, $\Delta\rho_{min}$ (e Å^{-3})	1.40, -1.18

1.4.4 [Ru(macro)(MTSO)(Cl)](OTf)(MeOH), [7](OTf)(MeOH)

All reflection intensities were measured at 110(2) K using a SuperNova diffractometer (equipped with Atlas detector) with Mo *K*α radiation (λ = 0.71073 Å) under the program CrysAlisPro (Version CrysAlisPro 1.171.39.29c, Rigaku OD, 2017). The same program was used to refine the cell dimensions and for data reduction. The structure was solved with the program SHELXS-2018/3 (Sheldrick, 2018) and was refined on F^2 with SHELXL-2018/3.[1] Numerical absorption correction based on gaussian integration over a multifaceted crystal model was applied using CrysAlisPro. The temperature of the data collection was controlled using the system Cryojet (manufactured by Oxford Instruments). The H atoms were placed at calculated positions using the instructions AFIX 43, AFIX 137 or AFIX 147 with isotropic displacement parameters having values 1.2 or 1.5 U_{eq} of the attached C, N or O atoms.

The structure is disordered. One part of the Ru complex and the triflate counterion are disordered over two orientations, and the occupancy factors of the major components of the disorder refine to 0.755(6) and 0.676(11), respectively. The asymmetric unit also contains one lattice MeOH solvent molecule that is found to be partially occupied (occupancy factor: 0.84(2)). The absolute configuration has been established by anomalous dispersion effects in diffraction measurements on the crystal, and the Flack and Hooft parameters refine to 0.039(15) and 0.054(13).

791_ALERT_4_G Model has Chirality at S1 (Sohnke SpGr) S Verify

Table S4. Crystallographic Data for [7](OTf)(MeOH)

	**[7](OTf)(MeOH)**
Crystal data	
Chemical formula	$C_{29}H_{26}ClN_6O_2RuS \cdot CF_3O_3S \cdot 0.84(CH_4O)$
M_r	835.12
Crystal system, space group	Orthorhombic, $P2_12_12_1$
Temperature (K)	110
a, *b*, *c* (Å)	13.6866 (3), 13.9815 (4), 17.2853 (5)
V ($Å^3$)	3307.70 (15)
Z	4
Radiation type	Mo *K*α
μ (mm^{-1})	0.75
Crystal size (mm)	0.34 × 0.32 × 0.27
Data collection	
Diffractometer	SuperNova, Dual, Cu at zero, Atlas
Absorption correction	Gaussian<br>*CrysAlis PRO* 1.171.40.67a (Rigaku Oxford Diffraction, 2019) Numerical absorption correction based on gaussian integration over a multifaceted crystal model Empirical absorption correction using spherical harmonics, implemented in SCALE3 ABSPACK scaling algorithm.
T_{min}, T_{max}	0.414, 1.000
No. of measured, independent and observed [$I > 2\sigma(I)$] reflections	38471, 7606, 6827
R_{int}	0.044
$(\sin \theta/\lambda)_{max}$ ($Å^{-1}$)	0.650
Refinement	
$R[F^2 > 2\sigma(F^2)]$, $wR(F^2)$, S	0.054, 0.146, 1.05
No. of reflections	7606
No. of parameters	792
No. of restraints	1392
H-atom treatment	H-atom parameters constrained
$\Delta\rho_{max}$, $\Delta\rho_{min}$ (e $Å^{-3}$)	0.79, -0.45
Absolute structure	Flack x determined using 2700 quotients [(I+)-(I-)]/[(I+)+(I-)] (Parsons, Flack and Wagner, Acta Cryst. B69 (2013) 249-259).
Absolute structure parameter	0.039 (15)

1.4.5 [Pd(OMe-bapbpy)](OTf)$_2$, [8](OTf)$_2$

All reflection intensities were measured at 110(2) K using a SuperNova diffractometer (equipped with Atlas detector) with Mo *K*α radiation (λ = 0.71073 Å) under the program CrysAlisPro (Version CrysAlisPro 1.171.39.29c, Rigaku OD, 2017). The same program was used to refine the cell dimensions and for data reduction. The structure was solved with the program SHELXS-2018/3 (Sheldrick, 2018) and was refined on F^2 with SHELXL-2018/3.[1] Numerical absorption correction based on gaussian integration over a multifaceted crystal model was applied using CrysAlisPro. The temperature of the data collection was controlled using the system Cryojet (manufactured by Oxford Instruments). The H atoms were placed at calculated positions (unless otherwise specified) using the instructions AFIX 43 or AFIX 137 with isotropic displacement parameters having values 1.2 or 1.5 U_{eq} of the attached C atoms. The H atoms attached to N2X and N5X (X = A, B) were found from difference Fourier maps, and their coordinates were refined pseudofreely using the DFIX instruction in order to keep the N–H bond distances within an acceptable range.

The structure is mostly ordered. Two triflate counterions are found to be disordered over two orientations, and the occupancy factors of the major components of the disorder refine to 0.60(2) and 0.781(13).

Table S5. Crystallographic Data for $[8](OTf)_2$

	$[8](OTf)_2$
Crystal data	
Chemical formula	$C_{22}H_{20}N_6O_2Pd·2(CF_3O_3S)$
M_r	804.98
Crystal system, space group	Monoclinic, $P2_1/c$
Temperature (K)	110
a, *b*, *c* (Å)	12.12622 (17), 15.5237 (2), 30.6528 (6)
β (°)	91.9453 (14)
V (Å^3)	5766.87 (16)
Z	8
Radiation type	Mo $K\alpha$
μ (mm^{-1})	0.89
Crystal size (mm)	0.56 × 0.13 × 0.10
Data collection	
Diffractometer	SuperNova, Dual, Cu at zero, Atlas
Absorption correction	Gaussian<br>*CrysAlis PRO* 1.171.40.67a (Rigaku Oxford Diffraction, 2019) Numerical absorption correction based on gaussian integration over a multifaceted crystal model Empirical absorption correction using spherical harmonics, implemented in SCALE3 ABSPACK scaling algorithm.
T_{min}, T_{max}	0.508, 1.000
No. of measured, independent and observed [$I > 2\sigma(I)$] reflections	55479, 13250, 12052
R_{int}	0.021
$(\sin \theta/\lambda)_{max}$ (Å^{-1})	0.650
Refinement	
$R[F^2 > 2\sigma(F^2)]$, $wR(F^2)$, S	0.036, 0.112, 1.04
No. of reflections	13250
No. of parameters	991
No. of restraints	498
H-atom treatment	H atoms treated by a mixture of independent and constrained refinement
	$w = 1/[\sigma^2(F_o^2) + (0.0601P)^2 + 15.2861P]$ where $P = (F_o^2 + 2F_c^2)/3$
$\Delta\rho_{max}$, $\Delta\rho_{min}$ (e Å^{-3})	0.84, -1.58

2 References

(1) Sheldrick, G. M. Crystal Structure Refinement with It SHELXL. *Acta Crystallogr. Sect. C* **2015**, *71* (1), 3–8. https://doi.org/10.1107/S2053229614024218.
(2) Spek, A. L. Structure Validation in Chemical Crystallography. *Acta Crystallogr. Sect. D* **2009**, *65* (2), 148–155. https://doi.org/10.1107/S090744490804362X.
